# Exploring the Links between the Quality of Early Mother-Infant Interactions and Child Physiological, Behavioural, and Developmental Outcomes. Do we Understand their Complexity?

**DOI:** 10.34763/jmotherandchild.20252901.d-25-00017

**Published:** 2025-09-10

**Authors:** Anna Grochowska, Grażyna Kmita

**Affiliations:** Interdisciplinary Doctoral School, University of Warsaw, Warsaw, Poland; Department of Psychology, University of Warsaw, Warsaw, Poland; Department of Early Psychological Intervention, Institute of Mother and Child, Warsaw, Poland

**Keywords:** early interaction quality, mother-infant relationship, reciprocity, dyadic synchrony, responsiveness, infant development, systematic review

## Abstract

Various aspects concerning the quality of mother-infant early interactions have been identified as developmentally supportive, promoting child socio-emotional and cognitive skills or related to less favourable outcomes. This review aims to: 1) an overview of observation-based measures assessing the quality of earliest mother-infant interactions, 2) systematically categorize associated behavioural and developmental outcomes, 3) identify potential gaps, together with promising new directions in research.

We conducted systematic searches of SCOPUS, Web of Science, PubMed, Cochrane Library, EBSCO, and Google Scholar, in which 30 articles met the eligibility criteria and were selected. Only empirical papers were included, if the quality of mother-infant interactions was measured by direct observation within the first 8 months of the infant’s life. The interaction quality measures were divided into unidirectional ones, focused on maternal contribution to the interaction, and bidirectional ones, describing mutual dynamics during interaction. We found that within the selected literature, unilateral methods were prevalent over the bilateral approach. What is more, we identified a research gap concerning the relationship between bidirectional indices of interaction quality and child physiological and neurodevelopmental outcomes. Further studies are needed to comprehend the cascading relationships, through which interactional experience modulates a baby’s physiological response, resulting in specific developmental outcomes.

## Introduction

The way mothers and infants communicate from the very beginning of their relationship is an important factor shaping a baby’s functioning and development, with important implications for infant mental health [ 1,2]. The early caregiving environment not only supports survival but also provides foundations for the infant’s cognitive, emotional, social, and regulatory abilities [3,4]. To understand how the early relational experiences may support certain changes in infants’ developmental trajectory, we have to look closer at the quality of interaction with a caregiver and identify particular interactive components that constitute the mutual sequential coordination patterns, what is called interactional affective-behavioural contingency [5].

### Theoretical approaches

Many studies highlight infants’ innate psychological abilities to adjust to their social surroundings and to express interest and attentiveness to the partner’s interactive behaviours. To maintain a sequentially contingent behavioural chain in the interaction with neonates, caregivers must match their activity level and coordinate it temporally [6,7]. Thus, initially, the infant’s interacting partner and their dynamic adaptation to the newborn’s signals were seen as a main factor modulating mutual communication and creating dyadic synchrony in parent-infant relationships [6]. In line with this view, adequate attunement between an infant and a parent, which means suitable adjustment to each other’s state, is primarily promoted via asymmetrical adjustments on the part of a caregiver. In this theoretical approach, the quality of caregiver-infant interaction is understood in terms of a continuum of parental observable behaviours that facilitate or inhibit the occurrence of dyadic synchrony [8] Parents need to present with sensitivity and responsiveness to appropriately read an infant’s subtle signs of ongoing readiness to interact or a burgeoning need to disengage [6,9].

In parallel, another approach can be observed, which emphasizes the active role of an infant and their behavioural and emotional contribution to communication in early social interactions with parents/adults. The newest approaches consider early parent-infant interaction as a reciprocal process of moment-to-moment social exchanges, where the infant is an active partner in dyadic communication, bringing their personal forms of intersubjectivity into their early relationships [4]. Following Stern (1971) [10], who was one of the first to conceptualize and analyse mutual, bidirectional social regulation between mother and infant, many researchers have pointed out the infants’ active role in co-constructing interactional interchange with a caregiver. Beebe and colleagues support bidirectional coordination dynamics between mother and infant in their research on interactive contingency [5]. In addition, they proved it to be an asymmetrical process, as mothers adjusted their behaviours to infants more than infants adjusted to their mothers [5]. Fogel’s co-regulation concept [11] captures relational communicative dynamics between mother and infant over time. Tronick’s mutual regulation model [12] highlights oscillating changes of synchrony in mother-infant communicative units. Finally, Feldman’s bio-behavioural synchrony model [13] integrates physiological and behavioural processes during the ongoing process of mother-infant interaction.

### Description of the methodology

During the last five decades, the above-mentioned theoretical approaches gave rise to diverse, established measures of assessing the quality of interaction between mother and infant, many of which helped to empirically prove the links between early interactional patterns and further child development [14,15]. For the purpose of this review, we report both unidirectional, as well as reciprocal, bidirectional approaches to the measurement of the quality of early caregiver-infant interaction.

As far as the former is concerned, the components of caregiver behaviour, which can impact dyadic synchrony, relate to different sensory modalities and may concern selection of time, form, or intensity of reaction. Evaluation of parenting quality based on sensory signals consists of gaze analysis, such as frequency and duration of looking to the infant versus no eye contact [16,17,18,19], the analysis of vocalizing to the infant [16,17,19,21], proportion and type of maternal touch [16,18] or sensory signal coordination, e.g., calculating of maternal predictability rate [15]. This article also reports parental communication qualities, centred on the study of valence of certain signals, such as maternal sensitivity [3,17,21,22,23,24,25,26,27], responsiveness [ 28,29,30,31,32,33,34], (), directiveness [32], intrusiveness [20,21,22,33], involvement [21], affect toward the infant [20,22,24,25,29,33,34,35,36], (or, so-called, mind-mindedness [33,34] (the exact meaning of each of the terms is explained in the Findings section). There are also studies where parental interactional qualities are summed up and categorized with reference to two overall domains: typical caregiving behaviour and atypical caregiving behaviour [35].

In contrast, the methodology based on bidirectional theoretical models captures reciprocal patterns of communication process over time. The researchers representing this approach examine the aspects of reciprocity [22,37,38,39,40], (synchrony in time series analysis [28,30,41,42], () or co-regulation sequences between interacting partners [43]. The better dyadic adaptation to each other’s needs, the more fluent is verbal and non-verbal social exchange, characterized by easy engagement, simultaneous movement, similarity, coordination, smoothness or gradual cool-down [28,39]. The quality of interaction here can also be clustered in two groups: good versus poor interaction regarding overall score of synchrony-related items [44].

### Possible explanation of interrelation and underlying mechanisms

In this review, we are looking for the after-effects of different levels of parent-infant attunement. There are several theoretical models explaining possible mechanisms of how early contact with the infant’s closest people modifies neuronal, physiological and genetical pathways resulting, subsequently, in individual constellation of social and behavioural abilities. Regulation theory [45] presents how earliest social stimuli may modulate production of neurohormones and possibly initiate a descending chain reaction, starting with mesocortical and dopaminergic pathways that communicate with the hypothalamic–pituitary–adrenal (HPA) axis and the sympathetic-adrenal-medullary (SAM) stress response system, ending with vagal autoregulation of the parasympathetic nervous system (PSN), which diminishes increasing arousal. Vagal PSN activity of an infant can, in turn, be supportive for synaptic growth and create an effective stress response system, thus building a child’s adaptive competences. All this means that social impulses of different qualities can influence social abilities from the earliest period of life.

### Why it is important to do this review

It is very important to evaluate where we are now in the context of the knowledge about adaptive patterns of mother-infant interactional behaviour and to identify the factors of highest impact on infants’ physiological pathways and evolving brain functions. By early identification of those factors, we could promote optimal stimulation for particular brain circuits, we could increase chances of proper newborn development, and avoid the risk of child’s psychological maladaptation.

### Main objective

In this systematic review, we explore the interrelation between different aspects of the quality of parent-infant early interactions and later functioning and development of the child. We considered the contribution effect of increasing the quality of early mother-infant interaction on biological and behavioural aspects of child functioning.

Selected papers had to be based on direct measurements of mother-infant interaction quality, and we organized them according to diverse methodological approaches, as well as different domains of children’s behavioural and developmental outcomes covering their social, cognitive, emotional, and neurophysiological functioning. With such a vast variety of data on interactional quality measuring methods as well as a diversity of children’s developmental outcomes, we were unable to perform a meta-analysis on the topic, and instead, we decided to conduct a systematic review.

Our analysis was guided by the following research questions: What aspects of early mother-infant interactions have been identified as factors promoting versus undermining child development? How was interactional quality operationalized? What aspects of child functioning and development have been most frequently versus rarely observed and related to the quality of the early mother-infant interaction?

## Materials and methods

### Criteria

The systematic review included articles reporting results of empirical research. All theoretical papers, book chapters, other reviews, and narrative dissertations were excluded. The considered papers had to be written in English and had to match the following inclusion criteria: The papers presented an application of a direct method of parent-infant interaction quality measurement, both at home or in a hospital setting. The interaction quality was assessed up to the eighth month of the infant’s corrected age (CA), i.e., before the advancements in cognitive, communicative, and motor development typical for the last quarter of the first year of a child’s life. The study presented correlation of mother-child interaction quality with early functioning or developmental outcomes of a child. Included papers must have referred to the neonates or infants without brain injuries, serious congenital conditions, anomalies or other risk factors of abnormal neurodevelopment (e.g., high-grade Intraventricular hemorrhage (IVH), hypoxic ischemic encephalopathy (HIE), cerebral palsy, leukomalacia, congenital heart disease (CHD), malformation syndromes, genetic diseases, severe metabolic defects, sensory deficits, intrauterine drug exposure, seropositivity, etc.).

### Search methods

**Figure 1.**
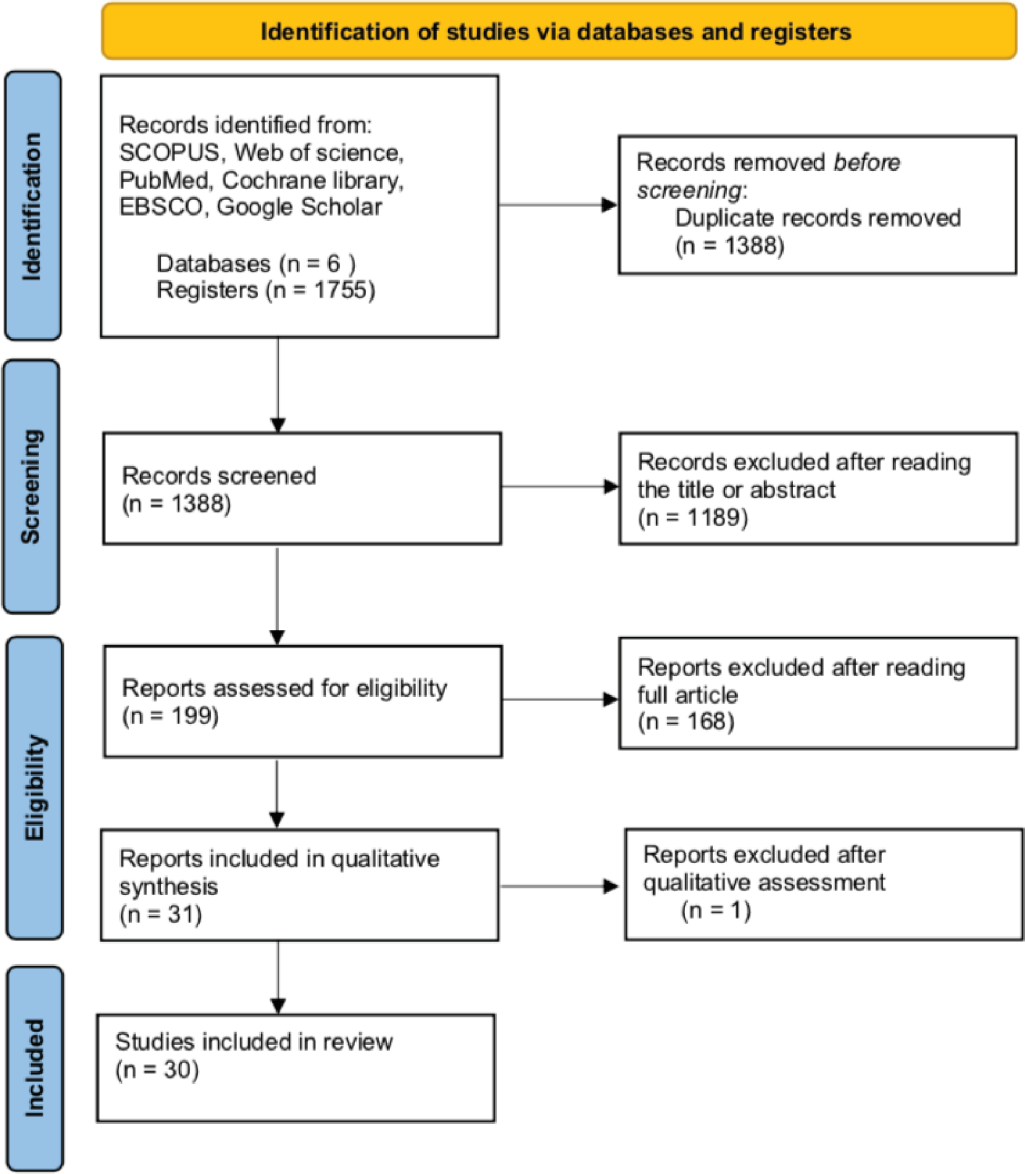
Systematic review flow diagram based on PRISMA guidelines.

The systematic search was pursued according to the PRISMA guidelines. (A flow chart of study selection is presented in Fig. 1). Six databases were used: SCOPUS, Web of Science, PubMed, Cochrane Library, EBSCO, and Google Scholar. The three domains of searching keywords were defined within the “(a)Who–(b)What–(c)How” scope, resulting in (a) newborn, preterm, premature, neonate, infant, (b) maternal care, parent-child interaction, parent- infant synchrony, attunement, maternal responsiveness, reciprocity, early relation (c)child development, developmental outcome, neurobehavioural outcome, early infancy, pain reactivity, autonomic nervous system, early temperament, stability, physiological response, physiological activity. The selected terms were diversified using medical subject headings (MeSH) terms and connected using Boolean operators.

We also would like to clarify why we chose to include keywords like “prematurity” in our literature search. Our decision was shaped by the fact that many studies exploring the very first stages of mother-infant interaction are conducted in neonatal intensive care units (NICUs), where preterm infants often remain hospitalized together with their mothers for extended periods. These circumstances create a unique setting in which early relational processes can be observed. Additionally, we also chose to include “autonomic reactivity”, which provides one of the few physiological tools available for capturing the subtle, reciprocal dynamics that emerge between a mother and her newborn in the earliest moments. The specific constellation of words used for each database can be found in Appendix 1.

The records were checked for duplicates using Mendeley software (Elsevier Inc.). Remaining papers were first screened for eligibility criteria by reading titles and abstracts and subsequently, filtered and selected by reviewing the full articles. The screening in databases resulted in finding 1,755 papers; 1,388 remained after eliminating the duplicates. The screening procedure led to the exclusion of most of the initially identified records. Out of the 1,388 retrieved articles, 199 were retained after the abstract screening in relation to predefined inclusion criteria. Then, after the full-text review process, 31 studies were ultimately included for quality assessment.

The quality assessment of selected papers was performed by two independent researchers by applying the Quality Assessment Tool for Quantitative Studies [46,47]. The level of concordance between the raters was 97%. The disagreements were judged and solved by the second author of this review. The general quality scores of papers were ranked between strong (15), moderate (15) and weak (1). The selection of right representation of the target population was mostly moderate, since mothers were usually asked to participate in the study with their children in a systematic way, in a clinical NICU environment. Study design was also largely ranked as moderate since the majority of studies were non-randomized cohort studies. Confounders’ control was not consistent among studies, fluctuating between strong, moderate, and weak. Each one of the studies presented a strong score of data collection method quality. Implementing a blind procedure across selected works was to a great extent ranked as moderate, since usually at least the participants were unaware of research questions. In several articles, participants’ withdrawals and dropouts during the study were not reported, so those studies were ranked as weak in this component score. The summary of the studies’ quality assessment included in this systematic review is attached in Appendix 2.

One study was excluded after quality assessment due to a weak overall quality score. The quality appraisal resulted in 30 records included in the final systematic review.

## Results

A comparison of the selected papers with these extracted data is presented in Table 1: author, year of publication, group sample, measures of interactional quality, domain of outcome, and main findings.

**Table 1.** List of studies included in the review.

**No.**	**Author / Year**	**Final sample (n)**	**Study design**	**Quality of interaction measures** Data collection method / *Infant’s age at measuring interaction*	**Outcome domain** Data collection method /*Child’s age at measuring the outcome*	**Main findings (↑= ↓ negative correlation, ↑= ↑ positive correlation)**
1.	A. Bernier, S. D. Calkins, M. A. Bell (2016)	n= 197 mother-infant dyads (healthy full-term infants)	Longitudinal study	**Maternal behavior: sensitivity, intrusiveness, positive affect, physical stimulation** coding scheme developed by Calkins, Hungerford, and Dedmon (2004)/ *5 months*	**Resting EEG power/** *5, 10 and 24 months*	↑ **Quality of maternal behavior during mother—infant interactions = ↑frontal resting EEG power**
2.	J. R. Britton, H. L. Britton, V. Gronwaldt (2006)	n= 152 mother-infant dyads (healthy full-term infants)	Longitudinal study	**Quality of the dyadic interaction scales** Nursing Child Assessment Satellite Training (NCAST) Feeding Scale/ *6 months*	**Security of infant-mother attachment.** Ainsworth Strange Situation Procedure/ *12 months*	**↑Quality of the dyadic interaction = ↑security of attachment**
3.	R. Costa, B. Figueiredo (2011)	n= 94 mother-infant dyads (7,2% preterm infants, 92,8% full-term infants)	Longitudinal study	**Quality of the overall mother–infant interaction (good vs poor)** The Global Rating Scales (GRS)/ *2 months (+/−5 days)*	**Temperament** The Infant Behavior Questionnaire - Revised (IBQ-R)- The Portuguese version/ *3 and 12 months*	**Poor mother–infant interaction = ↓mothers’ perception of infant high pleasure and smile and ↑ mothers’ perceptions of infant level of activity**
4.	J. S. Crawford (2004)	n= 65 mother-infant dyads (no information about GA)	Cross-sectional study	**Maternal responsivity/sensitivity Synchrony/reciprocity of parent-child interactions** 7-point Likert scales/ *between 6 and 12 months*	**Temperament.** The Infant Behavior Questionnaire- Revised (IBQ-R) and Temperament Laboratory Assessment (TLA)/ *between 6 and 12 months*	**↑Maternal responsivity/sensitivity = ↑child’s attentional skills (Perceptual sensitivity)**
5.	E. P. Davis et al. (2018)	Irvine Cohort: n_1_=192 mother-infant dyads Turku Cohort: n_2_=126 mother-infant dyads (infants selected from bigger “FinnBrain” Cohort, no information about GA)	Longitudinal cohort study	**Degree of predictability of maternal sensory signals** The entropy rate/ *6, 8 and 12 months*	**Child self-control** Infant Behavior Questionnaire - Revised (IBQ-R)/ *1 year*, Children’s Behavior Questionnaire (CBQ)/ *5 years,* Flanker task/ *6,5 years*, Temperament in Early Childhood Questionnaire (TMCQ)/ *9,5 years*	**↑ Unpredictable maternal sensory signals (high entropy rate) = ↓ child self-control**
6.	S. J. Erickson, J. R. Lowe (2008)	n= 50 mother-infant dyads (VLBW preterm infants: birth weight < 1,250 g.)	Cross-sectional study	**Interactive maternal behaviors** Still Face (SF) Tronick et al.’s (1978) protocol/ *between 6 and 8 months*	**infant positive and negative affect** Scale adapted from the Infant Regulatory Scoring System **infant development** BSID-II **infant temperament** Infant Behavior Questionnaire (IBQ) *between 6 and 8 months*	**Positive infant affect during and after the SF stressor was related to baseline infant positive affect and ↑ maternal responsiveness**
7.	C. A. Evans, C. L. Porter (2009)	n ranged from 101 to 84, mother–infant dyads (healthy full-term infants)	Longitudinal study	**Mother-infant co-regulation** Fogel’s Relational Coding System/ *6, 9 and 12 months*	**Infant developmental status: psychomotor (PDI) and mental (MDI) development** Bayley Scales of Infant Development (BSIDII)/ *6 and 9 months* **Attachment quality** Ainsworth Strange Situation Procedure/ *12 months*	**Symmetrical co-regulation = ↑ MDI scores. Asymmetrical and Unilateral co-regulation = ↓ MDI scores Symmetrical co-regulation = ↑PDI scores, Unengaged behaviors = ↓ PDI scores**
8.	R. Feldman (2007)	n= 31 mother-infant dyads (healthy full-term infants)	Longitudinal study	**Mother-infant synchrony. Mutual influence** assessed with time-series analysis (CCF cross corelation function)/ *3 and 9 months*	**Verbal IQ,** Stanford - Binet Inteligence Scale **Behavior problems,** The child Behavior Checklist (CBCL) **Child self-regulated compliance,** Observation at “do (pick up) or don’t (resist temptation)” paradigm **Maternal warm control discipline,** Maternal disciplinary style coded *I, II, III, IV: 2, 4, and 6 years* **Moral cognition,** 4 moral dilemmas **Dialogical empathy,** 2 conflict discusions *V, VI: 6 and 13 years*	**↑ Mother-infant synchrony — ↑ mutual influence, ↑ verbal IQ, ↑ behavior problems and ↑ capacity for empathy in adolescence, ↑ Mutual influence = ↑ self- regulated compliance and ↑ capacity for empathy in adolescence**
9.	R. Feldman (2010)	n= 36 mother-infant dyads (healthy full-term infants)	Longitudinal study	**Maternal sensitivity Child social engagement Mother intrusiveness Dyadic reciprocity** Coding Interactive behavior manual (CIB)/ *3 and 9 months, 2, 4, 6, and 13 years.*	**Intelligence** Stanford-Binet Intelligence Scale 4th Edition/ *2, 4 years* Wechsler Intelligence Test for Children - Revised (WISC-R)/ *6 years* **Infant difficult temperament** Infant Characteristic Questionnaire and Fussy-Difficult composite/ *3, 9, and 24 months* **Child behavior problems** Child Behavior Checklist 2–3 years (CBCL 2–3)/ *2 years* Child Behavior Checklist (CBCL)/ *4, 6 and 13 years* **Child depressive symptoms** Child Depression Inventory (CDI)/ *13 years*	**↓Maternal sensitivity across development and ↑ Intrusiveness = ↓ adolescents’ adaptation ↑Dyadic reciprocity = ↑ adolescent adjustment.**
10.	R. Feldman et al. (2013)	n= 50 mother /father -infant dyads (50 mothers, 48 fathers, healthy infants) f= 50 ‘best friends’ of a similar age to the target child	Longitudinal study	**Early Parental Care** Proportions of gaze to infant, positive affect, ‘motherese’ vocalizations, and affectionate touch were summed into a parent care composite/ *1 and 6 months*	**DNA** Genotyping **OT assessments** OT collection from Saliva/ *3 years* **Child’s Social Reciprocity: mother- child, father- child. Child with best friend** Coding Interactive Behavior manual (CIB)/ *3 years*	**↑Early maternal care = ↑ mother-child reciprocity (early paternal care was unrelated to father-child reciprocity) ↑Mother-child reciprocity = ↑ Children’s social reciprocity with best friend (father-child reciprocity was unrelated)**
11.	R. Feldman, E. Bamberger, Y. Kanat-Maymon (2013)	n ranged from 100 to 68; mother/father-infant dyads (healthy full-term infants)	Longitudinal study	**Parent-child reciprocity** Coding Interactive Behavior manual (CIB)/ *5 months, 3 and 13 years.*	**Children’s social competence,** **Aggression,** **Prosocial behavior** Nursery Assessment Scale *I, II, III 3 years* **Children’s dialogical skills** Adolescent-friend interactions/ *13 years*	**↑Early maternal and paternal reciprocity = ↑ social competence, ↓aggression in preschool, ↑dialogical skills in adolescence (Father—adolescent reciprocity contributed to the dialogical negotiation of conflict, whereas mother-adolescent reciprocity predicted adolescents’ dialogical skills during positive exchanges.)**
12.	R Feldman (2015)	n= 125 mother-infants dyads (healthy premature infants: GA between 25 and 35 weeks)	Longitudinal study	**Parent-child reciprocity** Coding Interactive Behavior manual (CIB)/ *3, 6, 12, and 24 months and 5 years*	**Emotion regulation (ER)** Age-specific ER paradigms/ *3, 6, 12, and 24 months and 5 years* **Autonomic regulation= RSA (vagal tone),** 10 min of baseline ECG **Child accident proneness,** child’s accident proneness and tendency for risky behavior on eight items **Child empathy,** Direct observations and an experimental paradigm **Behavior problems,** Child Behavior Checklist (CBCL) *II, III, IV, V at 10 years*	**↑ Parent-child reciprocity = ↑ child ER, ↑Reciprocity = ↑ greater empathy**
13.	C. Giovanelli et al. (2020)	n= 43 mother-infant dyads(no information about GA)	Longitudinal study	**Mind-mindedness** Meins and Fernyhough’s coding system (1977)/ *6 and 12 months*	**Symbolic play** The coding system developed by McCune-Nicolich (1977)/ *12 and 13 months*	**↑ Mind-related comments = ↑ length and maturity level of infants’ symbolic play**
14.	M. Kivijärvi et al. (2005)	n= 56 mother-infant dyads (healthy full-term infants)	Longitudinal study	**Maternal sensitivity behavior (MSB)** Parent-Child Early Relational Assessment (PCERA)/ *3 and 12 months*	**Temperament** PCERA infant items/ *3 months* Revised Infant Temperament Questionnaire (R-ITQ)/ *6 months* Toddler Temperament Questionnaire (TTQ)/ *12 months*	**Less sensitive (LS) mothers = ↑ infants of intermediate temperament cluster (68%)** **More sensitive (MS) mothers = ↓ infants of intermediate temperament cluster (35%)** **MS mothers = ↓ active infants (than those of LS mothers)** **LS mothers = ↑ infants with concern scores in Mood (than those of MS mothers)** **LS mothers = ↑ concern scores in Sociability of infants (than in case of MS mothers)**
15.	M. F. Lorber, B. Egeland (2011)	n ranged from 267 to 243, mother–child dyads (no information about GA, infants assessed at home at their 7^th^ day of life= probably full-term infants )	Longitudinal study	**Negative infancy parenting.** Ratings of positive and negative maternal regard for the infant on 7-point scales/ *3 and 6 months (common variance)*	**Maternal perception of infant difficulty.** The Infant Temperament Questionnaire (ITQ)/ *3 and 6 months* **Mutually negative mother–toddler interaction.** Series of mother teaching/child problem-solving tasks/ *24 and 42 months* **Conduct problems (CP)** Child Behavior Checklist (CBC)/ *at kindergarten and first grade (5–6 years)*	**↑Negative mothering = ↑mutually angry and hostile mother–toddler interaction, and ↑CPs**
16.	J. Milgrom et al. (2013)	m = 109 mothers with n=123 infants (preterm infants <30 weeks GA - Intervention group: m1= 54 mothers, n1 = 60 infants. Control group: m2= 55 mothers n2= 63 infants)	RCT (enhanced MITP intervention group vs. control group)	**Maternal sensitivity** Preterm Mother–Infant Interaction Scale (PREMIIS)/ *term-equivalent age*	**Infant communication abilities** The Infant–Toddler Checklist of the Communication and Symbolic Behaviour Scales Developmental Profile (CSBS DP Infant–Toddler Checklist)/ *6 months CA*	**Experimental (MITP intervention) group: ↑ symbolic behaviour**
17.	J. Milgrom, D. T. Westley, A. W. Gemmill (2004)	n ranged from 88 to 56, mother-infant dyads: n, ranged from 40 to 23 (depressed mothers), n_2_ ranged from 48 to 33 (control mothers), no information about infants’ GA	Cross-sectional study	**Maternal responsiveness** measure adapted by Milgrom and Burn (1988)/ *6 months*	**Temperament** The Short Temperament Scale for Infants (STSI) and Short Temperament Scale for Toddlers (STST)/ *12, 24 and 42 months* **Infant IQ** Wechsler Preschool Primary Scale of Intelligence (Revised)/ *42 months* **Infant cognition** Early Screening Profiles (ESP)/ *42 months*	**Depressed mothers group: ↓ mother-infant interactions and ↑ child’s temperamental difficulties (but not correlated with maternal responsiveness) ↓Maternal responsiveness = ↑ cognitive deficits**
18.	C A, Newnham, J. Milgrom, H. Skouteris (2009)	n= 68 mother-infant dyads (preterm infants; Intervention group: n1= 35 (mean GA= 31.26), Control group: n2= 33 (mean GA= 33.71)	RCT (MITP-based intervention group vs control group)	**Maternal respond, Infant alert, Attending to mother. Mutual attention, Reciprocity/Synchrony** Synchrony Scale/ *3 and 6 months CA*	**Temperament and the colic, sleep and excessive crying difficulties** Short Temperament Scale for Infants. The 7-item Approach subscale of the STSI/ *3 and 6 months* **Communication and Problem Solving.** The Ages and Stages Questionnaire (ASQ)/ *2 years*	**Experimental (MITP intervention) group: Infants were perceived as more “approaching”, “easier” ↓Fewer colic, ↓Sleep and crying difficulties ↑Communication (ASQ)** **↑Maternal Responsiveness and Infant Alertness = ↑Communication**
19.	K. A. O’Donnell H. Gaudreau (2014)	n ranged from 213 to 109, mother- infant dyads (healthy full-term infants)	Longitudinal study	**Maternal sensitivity** Ainsworth Maternal Sensitivity Scales *6 and 18 months* **Maternal behavior: duration of looking away from the infant, vocalizing to the infant** Behavioural Evaluation Strategies and Taxonomies coding system/ *6 months*	**Children’s attachment security** Modified Strange Situation procedure/ *36 months*	**No correlation was found between maternal sensitivity or maternal behavior and children’s attachment security.**
20.	J Poehlman n, B. H. Fiese (2001)	n= 84 infant-mother dyads (n_1_ =44 full-term infants, n_2_= 20 preterm LBW infants <2500 g, n_3_= 20 preterm VLBW infants < 1500 g )	Longitudinal cohort study	**Quality of parent-infant interaction** The Pediatric Infant Parent Exam (PIPE)/ *6 months*	**Infant developmental abilities** The Mental Scale (MDI) of the Bayley Scales of Infant Development/ *12 months*	**↑ Reciprocal, ↑affectively positive and ↑engaging interactions = ↑ Infant developmental abilities**
21.	V. Sethna et al. (2017)	n= 39 mother-infant dyads (healthy full-term infants)	Cross-sectional study	**Maternal sensitivity and affect, Quality of parent-infant relationship, Infant communication and affective state** Global Rating Scales (GRS)/ *between 3 and 6 months*	**Dimensions and brain volumes** MRI data acquisition/ *between 3 and 6 months*	**↑Maternal affect = ↑total grey and white matter volume, and ↓CSF volume** **↑Maternal sensitivity = ↑subcortical grey matter volume** **↑ Infant communication and engagement during mother-infant interactions = ↓cerebellar volume**
22.	P. E, Shah, et al. (2013)	n= 123 mother-infant dyads (n_1_ = 39 very preterm infants (VPI ), n_2_= 47 moderate preterm infants (MPI), n_3_=37 late preterm infants (LPI)	Longitudinal study	**Maternal parenting** Parent Child Early Relational Assessment (PCERA)/ *4, 9, 16, and 24 months*	**Cognitive skills** Abbreviated Battery IQ Scale (ABIQ) from the Stanford-Binet Intelligence Scales, 5th edition **Child behavior problems** Child Behavior Checklist (CBCL) *both at 36 months*	**↑Negative parenting = ↑ externalizing behavior problems ↑Maternal negative behavior = ↓ optimal child IQ (VPIs). ↓ Negative interactions during infancy = ↑ cognitive skills (VPIs)**
23.	D. Silberstein et al. (2009)	n= 76 mother-infant dyads (low-risk premature infants: mean GA of 32.5 weeks)	Longitudinal study	**Maternal touch and gaze Maternal adaptation** Coding of Interactive Behavior-Newborn (CIB)/ *Prior to hospital discharge* **Infant developmental status: psychomotor (PDI) and mental (MDI) development** Bayley Scales of Infant Development, 2nd edition/ *4 months*	**I. Feeding difficulties** Maternal interview and direct observations of feeding interactions/ *1 year*	**↑Maternal intrusiveness, ↓affectionate touch and gaze, ↑gaze aversion and ↓maternal adaptation = ↑feeding difficulties**
24.	S. Stolt et al. (2014)	n= 62 mother-infant dyads (n_1_=28 VLBW infants = birth weight ≤ 1500 g, n_2_= 34 full-term infants )	Longitudinal cohort study	**Quality of mother-child Interaction scales** The Parent-child Early Relational Assessment (the PCERA)/ *6 and 12 months (CA for VLBW infants)*	**Early language development** Checklist for the Development of Early Vocalizations (CDEV) + Finnish version of the MacArthur Communicative Development Inventory (CDI; FinCDI)/ *6, 12 and 24 month (CA for VLBW infants)*	**↑ Maternal positive affective involvement = ↑number of morphological inflections ↑Infants positive affect = ↑number of morphological inflections ↑ Maternal positive communication = ↑language skills ↑Dyadic features = ↑language skills**
25.	M. T. Tu et al. (2007)	n= 158 mother-infant dyads (n_1_= 103 VLGA infants= ≤32 weeks GA, n_2_=55 full-term infants )	Longitudinal cohort study	**Interactive maternal behaviors: Gratification, Affect, Sensitivity, and Organization.** criteria set by Crnic et al. (1983)/ *8 months CA*	**Focused attention** Methods of Lawson and Ruff (2001) / *8 months CA* **Infant Cortisol Analysis** **Parenting stress** Long-form Parenting Stress Index (PSI) / *8 months CA*	**↑Interactive maternal behavior = ↑focused attention (preterm/low parenting stress group) ↑Interactive maternal behavior = ↓basal cortisol and ↓ quality of focused attention (preterm/high parenting stress group)**
26.	E. van de Weijer-Bergsma et al. (2016)	n= 74 mother-infant dyads (preterm infants; ≤36 weeks GA with a birth weight of <2500g)	Longitudinal study	**Maternal sensitive responsiveness and directiveness** ELO scales/ *7, 10 and 14 months CA*	**Executive functioning** looking and reaching versions of the A-not-B task/ *7, 10 and 14 months CA*	**↑ Maternal directiveness and stability in maternal directiveness = ↑ (faster) developmental change in A-not-B performance**
27.	F. F. Warnock et al. (2016)	n= 24 mother-infant dyads (healthy full-term infants)	Cross-sectional study	**Maternal caregiving behavior** Maternal Behavior Coding System (MBCS)/ *second visit after hospital discharge*	**Infant pain behavior self-regulation** Neonatal Distress Pain Related Behavioral Coding Schema (ND-BSC)/ *second visit after hospital discharge*	**Atypical maternal caregiving behavior was related to atypical infant pain behavior self-regulation during and after the heel lance procedure.**
28.	A. Wazana et al. (2015)	n= 650 mother-infant dyads (healthy full-term infants)	Longitudinal study	**Maternal sensitivity** Ainsworth Maternal Sensitivity Scales **Maternal behavior: frequency and duration of looking away from the infant, vocalizing to the infant, instrumental caregiving** Behavioural Evaluation Strategies and Taxonomies coding system/ *Both 6 months*	**Infant developmental status: psychomotor (PDI) and mental (MDI) development** The Bayley Scales of Infant Development—Second Edition/ *6, 12, and 18 months* **DRD4 genotype.** Genotyping **Attachment** The modified separation–reunion procedure/ *36 months*	**↑ Attentive maternal care (frequency of maternal looking away behavior) and ↓ Sensitivity = disorganized attachment of children in the midrange of birth weight. The association reversed with extreme birth weight (low and high).**
29.	D. Wolke, S. Eryigit-Madzwamuse, T. Gutbrod (2014)	n=176 mother-infant dyads; n_1_= 71 VP/VLBW infants: <1500 g or <32 weeks of gestation), n_2_= 105 full-term infants	Longitudinal cohort study	**Maternal sensitivity** Boston City Hospital Assessment of Parental Sensitivity (BCHAPS)/ *at term* Mother-Infant Structured Play Assessment/ *3 months*	**Infant attachment** Strange Situation Assessment (SSA)/ *18 months*	**↓ Maternal sensitivity at term (full-term sample) = ↑ attachment disorganization**
30.	M. A. J. Zeegers et al. (2018)	n ranged from 135 to 130 infants; mother/father – infant dyads (healthy infants = birth weight > 2500 g)	Longitudinal study	**Parental mind-mindedness** Mind-mindedness coding manual *4 and 12 months* **Parenting quality** Meso Behavioural Rating System for Families with young children (MeBRF)/ *12 months*	**Baseline HRV** Mean value of HRV **HRV decline** Stranger approach task *Both at 4 and 12 months*	**Both mothers’ and fathers’ appropriate mind-related comments = ↑infant baseline HRV (for fathers indirect association via parenting quality) Mothers’ appropriate mind-related comments and fathers’ non-attuned mind-related comments = ↑ HRV decline during the stranger approach**

### Preliminary analysis–characteristics of the included studies

#### Study design

Twenty-three articles had a longitudinal study design (76,7%), two were randomized control trials (6,7%) and five represented a cross-sectional approach (16,7%).

#### Sample

Among the papers, sample size of children and parent dyads ranged from 24 to 650).

Three studies (10%) included both mother and father interactions with an infant, but the majority (90%) presented observations of mother-infant dyads.

The findings of 13 papers (43,3%) related to healthy infants born full-term. Seven studies (23,3%) concerned preterm children, of which six included groups of very low birth-weight infants (VLBW < 1500 g) or very preterm infants (VPI < 32 weeks of GA). Five papers (16,7%) included both full-term and preterm groups. In five articles (16,7%), gestational age of infants was not clearly indicated.

In four studies, the direct observation and evaluation of the interaction quality was performed in the NICU prior to the hospital discharge. In one study, interactional measurements were taken when infants were 1 month old, and one when infants were 2 months old. Seven of the studies made their interaction quality assessment when infants were 3 months old. Three articles included the interactional measures performed when infants were 4 months old, and two when infants were 5 months old. Thirteen studies evaluated infant interactions when they were 6 months old and one when they were 7 months old. In two of the studies dyadic interactions were assessed by the end of the eighth month postpartum. The period of interactional assessment in two studies was defined as between 6 and 8 months), and in another one between 6 and 12 months).

As far as child developmental outcomes are concerned, 25 studies (83,3%) included developmental assessment in infancy (up to 12 months); 16 (53,3%) in toddlerhood (13–36 months) ; seven (23,3%) included preschool years (4–5 years); in five studies (16,7%) developmental assessment was implemented in school years (6–12 years) (; and just three of the studies (10%) used data from subjects’ adolescence (older than 12 years) in the analyses.

### Findings: Conceptualization and measurements of interactional quality

The interactional quality measures applied in the studies under this review were divided into two subgroups: (a) unidirectional indices of maternal features that contribute to general mother-infant communication and (b) bidirectional indices of mutual dynamics that appear in the early relationship. In this section, we only briefly delineate developmental outcomes correlated with specific interaction quality indices, as they will be elaborated on later in the text.

#### Unidirectional assessment of maternal signals

Maternal response constitutes a major part of infant early interactional experience [48]. Various features of maternal signals were evaluated as a measure of interactional quality in 23 papers (76,7%) finally included in this review.

#### Overall score of parenting quality

While the majority of researchers examined selected features of parental behaviour in interaction (listed below) and correlated behaviour with infant developmental outcome, a few took an overall perspective on measuring the caregiving quality.

Warnock and colleagues [35] used a microanalytical behavioural tool, called Maternal Behavior Coding System (MBCS) in order to distinguish two general domains of caregiving behaviour: typical behaviour (sensitive and responsive caregiving actions and interactions) from atypical behavious (“being less or overly responsive to their infant cues, intrusive, or they withdraw from interacting with their infants”). Mothers’ reactions were recorded during an infant pain event at NICU, coded and categorized to those two clusters. The results showed that atypical maternal caregiving behaviour was related to atypical infant self-regulation behaviour during and after a stressful event.

Tu and colleagues [26], on the other hand, distinguished “interactive maternal behavior” as a single factor score, calculated on the basis of Crnic’s coding criteria [49], which assessed gratification, affect, sensitivity, and organization in mothers’ behaviour during the session of free play with their infant. They found that the interactive maternal behaviour is correlated with focused attention and infant basal cortisol level (more details on the outcomes in the section Findings: Developmental outcome).

Feldman with colleagues (2013) used her original construct of “early parental care”, which summarized the average of the typical parental care behaviours, such as gaze to infant, positive affect, “motherese” vocalizations, and affectionate touch, measured during free interactions at 1 and 6 months of the infant’s age. The above-mentioned composite index of early parental care describes the proportion of time out of the entire session the parent engaged in caregiving behaviours, and it reflects the quality of early parenting. Child developmental outcome measured in the study was social reciprocity level. Researchers proved that early caregiving experience, together with parents’ oxytocin level (which was also controlled in the study) were factors that jointly explained the child’s social reciprocity in multiple relationships.

Shah and colleagues [21] evaluated maternal caregiving quality on the continuum from more positive to more negative, according to the general sum of scores in the Parent-Child Early Relational Assessment (PCERA) coding system [50].. Points were awarded in three parenting domains: (1) positive affect, involvement, and verbalizations; (2) negative affect and behaviour; and (3) intrusiveness, insensitivity, and inconsistency, and were added together to create the total parenting quality score. After correlating this variable with child behavioural problems and cognitive skills, the authors showed that more negative parenting was a predictor of externalizing behaviour problems and lower IQ.

Likewise, Zeegers and colleagues [33] calculated the general parenting quality index, based on four scales: responsiveness, intrusiveness (reversed), warmth/affectivity, and negativity (reversed). They applied the Meso Behavioural Rating System for Families (MeBRF) with young children [51] to code parental behaviour and affect during parent-infant free play. No statistically significant correlation of parenting quality with physiological regulation as an infant developmental outcome was found.

#### Maternal level of sensitivity/responsiveness

Maternal sensitivity concept derives from Ainsworth’s works, and it stands for the capability to receive infant signals, to process, interpret them correctly, and respond adequately and in a timely manner [52]., which creates the proper infant relational experiences. In order to assess the level of maternal sensitivity two research groups [17,19] (applied a gold standard measure, i.e., the macro-analytic Ainsworth Maternal Sensitivity Scales [52]. The tool consists of four subscales of maternal characteristics: acceptance, availability, cooperation, and sensitivity. One group [19] proved that less sensitive maternal behaviours can predict a disorganized type of attachment [53]; however, another one [17] did not find any significant correlation between maternal sensitivity and child’s security of attachment. In addition, Wolke and colleagues in their paper [27] reported significantly higher levels of attachment disorganization in 1.5-year-toddlers of mothers with lower sensitivity measured at the hospital unit, close after delivery, using the Boston City Hospital Assessment of Parental Sensitivity (BCHAPS) [54]. The tool was completed by nurses on the basis of their observation of maternal behaviours, and consisted of 12 items, which evaluated mothers’ caring, interacting, understanding, and enjoyment with their infant. At 3 months of infants’ age, the same team of researchers applied two different measures of maternal sensitivity, i.e., the Mother-Infant Structured Play Assessment, adapted from the Emotional Availability Scales [55] and Infant and Caregiver Engagement Phases [56]. Interestingly, they were not significantly correlated with attachment patterns, and only BCHAPS remained significantly related.

Another approach to measuring maternal sensitivity was presented by Milgrom with colleagues [29] and Newnham with his research group [30] in their randomized controlled trials. They implemented the Mother–Infant Transaction Program (MITP) intervention [57], which teaches mothers to recognize and minimize stress responses in preterm infants. After intervention, mothers in the experimental group [30] were more responsive to their infants than control mothers when assessed with the Synchrony Coding Scales [58]. The Maternal Respond Item measured the level of sensitivity in mothers’ responses to infant cues. Milgrom and colleagues [29] applied the same intervention and proved that it can successfully enhance the level of maternal sensitivity in the experimental group. They used the Preterm Mother–Infant Interaction Scale (PREMIIS), based on Synchrony Scales [58] to assess it. Maternal sensitive behaviour in this tool represents better awareness of infant negative cues, lower level of stressful impact on infants, and more appropriate caregiving. Both Milgrom’s and Newnham’s research groups found a positive correlation between maternal sensitivity and infant communication abilities.

Kivijärvi and colleagues [24] explored the construct of maternal sensitivity behaviour and its possible longitudinal consequences, namely, infant temperament. They implemented the PCERA Scale developed by Clark [50] to assess seven maternal interactional features: (1) enjoyment/pleasure, (2) contingent responsivity to child’s positive or age-appropriate behaviour, (3) contingent responsivity to child’s negative or unresponsive behaviour, (4) structures and mediates environment, (5) parent reads child’s cues and responds sensitively and appropriately, (6) connectedness, and (7) mirroring. The mothers who had the so-called “concern scores” on any of the above-mentioned items were categorized as the Less Sensitive (LS) mothers and those without any “concern score” were categorized as the More Sensitive (MS) mothers. The results point to interrelation between maternal sensitivity behaviour and infant temperament with potentially bidirectional links. Apparently, 12-month-old infants of LS mothers belonged mostly to the intermediate temperamental cluster, while this was true for only 35% of MS mothers’ infants. In addition, there were more infants of LS mothers with temperamental “concern scores” in mood than those of MS mothers.

Also, Sethna and colleagues, in their research [25], considered maternal sensitivity as an important factor related to infant development; they found a positive correlation of moderate effect size between maternal sensitivity and infant’s subcortical grey matter volume. The maternal sensitivity here was understood as warmth, acceptance, non-demanding, and non-intrusive behaviour; it was measured using the Global Rating Scales (GRS) [59].

Another closely related concept is maternal responsiveness, which is sometimes used interchangeably with maternal sensitivity. Although some researchers distinguish the two as separate constructs, others point out to the fact that it might be difficult to theoretically differentiate sensitivity from responsiveness [28]. Therefore, in this section, we present studies that also include maternal responsiveness defined as “a sensitivity in responding to infants’ cues” [34]. The more responsive a mother is, the more attentively she will notice and react to the subtle, minimal verbal or non-verbal cues. Milgrom and colleagues rated maternal responsiveness by frequency of its occurrence (0: < 30% of the time; 1: 30–60% of the time; and 2: > 60% of the time) during free interactions comparing two groups of depressed mothers and a control group of healthy mothers. Afterwards, they correlated maternal responsiveness with children’s cognitive competences and their temperamental profiles. The statistical modelling that authors used revealed that cognitive deficits in children of depressed mothers can be explained by lowered maternal responsiveness. No such mediating effect of maternal responsiveness was found, as far as children’s temperamental changes were concerned.

Erickson and Lowe [60] examined a level of maternal responsiveness using Haley and Stansbury’s coding system [61], including variables such as: (a) watching; (b) attention-seeking; (c) Mirror A (i.e., mother mimics infant’s behaviour in an exaggerated fashion); and (d) Mirror B (i.e., mother mimics infant’s behaviour; in return, the infant responds to the mother’s response in a positive manner). In their research, maternal responsiveness predicted the child’s baseline positive affect, and positive affect during and after an interactive stressor (more details on these developmental outcomes in the section Findings: Developmental outcome).

Crawford, in her research [28], assessed macroscopically maternal sensitivity/responsiveness coding parent-child free interactions according to a 7-point Likert scale (1 = extremely non-responsive/non-sensitive; 4 = moderately responsive/sensitive; 7 = extremely responsive/sensitive). It correlated with infant attentional skills. (More details on these developmental outcomes are included in the section Findings: Developmental outcome).

Other researchers [32] tried to measure differently sensitive responsiveness by using the ELO scales (Dutch Experimental Longitudinal Project; ELO) [62], and they connected results with the speed of development in infant executive functioning; however, they were not able to find significant effects.

#### Maternal level of directiveness vs. intrusiveness

Another two qualities of parenting appear to be seemingly close to each other but require a clear distinction because of their opposite effects on infant-caregiver interactions. The first one is positive parental directiveness, namely, “the degree to which a parent selects topics of conversation or play, uses imperatives and prompts to control or regulate the child’s attention or behavior” [32] that enriches the baby’s experience and supports their developmental outcomes. Van de Weijer-Bergsma with colleagues [32] used the ELO scales [62] to assess the level of maternal directiveness in play sessions and proved that directive mothers’ behaviour was positively related to faster developmental change of infants’ executive functions over time.

The second mentioned parenting quality is maternal intrusiveness, which in contrast to directiveness, seems to impair infant developmental outcome. It may include different kinds of behaviours: not only intrusive physical interactions, such as forcing toys or self on infant, but also overcontrolling reactions, failing to modulate own behaviour when the infant withdraws or turns away from interaction or higher focus on own agenda, while ignoring the infant’s cues [22]. Bernier and colleagues [22] scored the prevalence of those behaviours in early mother-infant interactions using a particular coding scheme [64] and correlated it with neurodevelopmental outcomes of infants, but the results showed no interrelation. However, another group of researchers [18] found a positive correlation between maternal intrusiveness and infants’ longitudinal outcome at 12 months of age, i.e., feeding difficulties. Maternal intrusiveness was observed during both feeding and non-feeding mother-child interactions and coded using the Coding of Interactive Behavior-Newborn (CIB) scale [65].

#### Affect toward the infant

A mother can display positive affection to her child in early interactions by cuddling and cooing or present negative affect towards an infant such as expressing anger, frustration, or disgust. There is evidence of a long-term intercorrelation between the valence of maternal affect and child developmental outcome [20,22]. Lorber and colleagues [36], in their longitudinal study, rated the mother’s expression of positive and negative regard during feeding in infants’ first months of life and found out that negative parenting variables predicted conduct problems in children during preschool age (5–6 years).

Bernier [22] proved that maternal positive affect, here coded as positive emotions expressed through the tone of voice or facial expressions, are positively related to infants’ higher frontal resting EEG power, which reflects brain development (further details below). However, Sethna’s results [25] showed that maternal affective state, here characterized by the level of maternal enjoyment, effort, and vitality, degree of self-consciousness, and the extent of anxiety in the interaction and expressions of negative affect (i.e., depressive-like statement), measured with the GRS [59], was significantly related to infants’ total grey and white matter volume, and CSF volume (more details on these developmental outcomes in the section Findings: Developmental outcome).

#### Touch and gaze

Silberstein and colleagues [18] microcoded maternal behaviour during feeding and non-feeding interaction with their infants using the CIB scale [65], which distinguishes the following behavioural attributes related to mother’s touch: (1) affectionate (kissing, caressing, hugging, gently stroking, or mother touching infant with clear positive affect); (2) functional (wiping mouth, arranging clothes or blanket); and (3) no touch (mother just holds baby), as well as related to mother’s gaze: (1) to the infant; (2) avert to bottle (i.e., mother averts her gaze from infant to look at the bottle); and (3) gaze aversion (no eye contact with infant or bottle). Subsequently, researchers correlated mothers’ touch and gaze in the neonatal period with the occurrence of feeding problems at the infant age of 12 months. The results showed that feeding difficulties were associated with less maternal affectionate touch and gaze during non-feeding interactions and more gaze aversion during feeding interactions.

#### Mind-mindedness

This construct [63] refers to the caregiver’s tendency of interpreting an infant’s behaviour in terms of the underlying mental processes, such as desires, thoughts, preferences and feelings, rather than an individual with needs that must be satisfied. Mind-mindedness (MM) is measured by observation of a parent’s verbal expressions towards their infants. High scores of MM reflect a parent’s capacity to make appropriate mind-related comments that accurately describe an infant’s internal states. Zeegers in 2018 [33] and Giovanelli with his colleagues [34] in 2020 used this theoretical concept to differentiate parental interactional competences and correlate them with infants’ developmental outcome. In their research, they assessed MM according to Meins and Fernyhough’s coding instruction [66], and found a positive correlation between mothers’ MM and average length and level of infants’ symbolic play in toddlerhood [34]. Zeegers and colleagues [33] found an independent association between mothers’ and fathers’ appropriate mind-related comments and infants’ higher HRV baseline index, which reflects better physiological regulation. In addition, mothers’ and fathers’ non-attuned comments independently contributed to worse physiological regulation.

#### Maternal dynamic patterns of multimodal interactive behaviour

Apart from established quantitative methods centred on measuring the valence of maternal characteristics in interaction, there are studies that searched for more overall patterns capturing mothers’ behaviours. Davis and colleagues [15] looked for sequences of maternal caregiving by coding the process of transitioning between auditory, visual, or tactile sensory stimuli. Researchers estimated the degree of probability of mothers’ behaviour by counting the number of possible combinations of subsequent sensory signals expressed to the child. They compared this entropy rate between mothers and correlated it with self-regulation of infants in their first postnatal months. Results showed that the more unpredictable maternal signals, the poorer self-regulation in infancy.

The observed maternal behaviour can be coded according to duration, form, and intensity of specific sensory signals and can relate to parental interactional qualities, such as sharing attention and vocalization. Wazana and colleagues [19] used the microanalytic Behavioral Evaluation Strategies and Taxonomies coding system to measure maternal sensory events, namely, frequency and duration of looking away from the infant and characteristics of vocalization to the infant (quiet talk, motherese, singing, and adult talk). It occurred that less attentive maternal care measured with higher frequency of looking away from infant behaviours was related to a disorganized style of attachment.

### Measurement of bidirectional dynamics

In the review, we identified 11 (36,7%) examples of the mutual mother-infant interaction evaluation methods. There were several different methodologies applied to capture bidirectional dynamics. Some of the tools first evaluate separately maternal, infantile, and dyadic interactional factors and summarize them to represent the overall, bidirectional quality of interaction. Others only include dyadic qualities of interactions, such as reciprocity, disorganization, co-regulation, level of synchrony, etc.

#### Scales reflecting the quality of bidirectional interaction

Britton and Gronwaldt [38] used the Nursing Child Assessment Satellite Training Feeding Scale [67] to probe the quality of mutual mother-infant interactions during the ongoing process of feeding infants by their mothers at 6 months postpartum. This tool is based on Barnard’s Parent/Caregiver-Child Interaction theoretical model, which describes the interactive system as a contingency of signals from a child to caregiver, and from caregiver to the child, since they constantly influence and modify each other’s behaviours, which makes the whole system acquire new properties [68]. In the above-mentioned interactional scales, the following factors are assessed: child’s clarity of clues and responsiveness, caregiver’s alleviation of distress and sensitivity to cues, and also environmental characteristics referring to both animate and inanimate elements. Britton and coworkers [38] found that a higher quality of bidirectional interactions, scored on previously mentioned scales, predicts an infant’s secure attachment at 12 months of age.

Stolt et al. [40] used the PCERA method to assess the quality of mother-child interaction at the same age (6 months). PCERA [50] is based on clinical observations and developmental theories and was meant to capture and analyse a child’s experience of the mother, and vice versa. The scales reflected three maternal, three infant, and three dyadic features, namely, dyadic mutuality, flatness, disorganization, and tension. These dyadic factors correlated positively with later linguistic competences of children measured at 12 and 24 months of age.

Poehlmann and Fiese [39] assessed quality of parent-infant interaction macro-analytically, using an observational rating of early parent-child interactions, called the Pediatric Infant Parent Exam (PIPE) [69]. This tool is designed to assess the degree of early interactional reciprocity and positive affect during interactional games. As a potentially related outcome, infant mental abilities were measured and the PIPE scores significantly correlated with infants’ mental development (further details below).

In addition, the already mentioned CIB manual, elaborated by Feldman (1998) [65], measures several aspects of mother-infant communication during free play, including dyadic reciprocity [16,22,37]. It addresses “the formal features of the interaction and describes an exchange where the dyad is moving in harmony, each is engaged and contributes to the mutual exchange, the interaction is the end product of the input of both partners, and the atmosphere is one of collaboration and joint activity, whether the activity is verbal or non-verbal, focuses on social give-and-receive or on object manipulation” [42], and it is calculated as an average of selected codes related to verbal and non-verbal signals, expressions, exchanges in gaze, smiles, gestures, breathing, dyadic tension, and mutual adaptation [23,37,42]. In 2010, Feldman correlated dyadic reciprocity in early mother-infant interactions with the data on the participant’s psychological adaptation across development until 13 years of age [22]. Her findings show that CIB’s dyadic reciprocity construct is a meaningful independent predictor of adolescents’ adaptation. In her later study [42], Feldman and colleagues explored both father-infant and mother-infant dyadic reciprocity and correlated it with children’s social competence and aggression. They found strong evidence that reciprocity contributes to lower aggression and better social skills (more details in the developmental outcome section). In 2015, Feldman [37] also discovered that dyadic reciprocity is a meaningful factor for emotion regulation and again, empathy.

Costa and Figueiredo [44] applied in their research the GRS [70], which probe the nature of engagement between mother and infant during their interaction. The total score on five assessed items (smooth/easy versus difficult; fun versus serious; satisfying versus unsatisfying; much engagement versus no engagement; excited engagement versus quiet engagement) results in two categories: good interaction cluster and poor interaction cluster. Authors Costa and Figueiredo [44] found that mother-infant interactional quality, measured this way, was associated with the infant’s temperamental profile (details below).

#### Synchrony measurements

The above-mentioned scales reflect some interactional dyadic factors that can be meaningful for the quality of interaction; however, some researchers tried to capture the dynamics of communication sequences over time.

Feldman’s approach [41] was to microcode mother’s and infant’s affective states in 1 s frames and assess synchrony of reactions with a time-series analysis (CCF; cross-correlation function). Feldman was searching for its contribution to specific child competences across 13 years of life, i.e., verbal IQ, child self-regulated compliance, behaviour problems, moral cognition, and dialogical empathy. Mother-infant synchrony across the first year appeared to be a significant predictor of some developmental outcomes, such as IQ, behaviour adaptation, and empathy (details below).

Crawford, in her doctoral dissertation [28], measured synchrony/reciprocity in mother-infant interactions by using a 7-point Likert scale, based on the work of Bemieri, Reznick, and Rosenthal (1988). A global rating was assigned to parent-child interactional behaviour by assessing three aspects of synchrony: simultaneous movement, tempo similarity, and coordination/smoothness. The author tried to relate the synchrony scores to follow up on child attentional skills; however, no significant interrelations were found.

#### Mutual co-regulation

Evans and Porter [43] observed the communication structure in mother-infant interactions, regardless of emotional content of the communicated message, looking for co-regulated sequences. They used Fogel’s Global Relational Coding System [71] in order to code co-regulation patterns of four dimensions: symmetrical, asymmetrical, unilateral, and disruptive, or a dimension of non-regulation or unengaged behaviours. Subsequently, they tried to correlate these dimensions with mental and psychomotor developmental scores and attachment style. Findings showed that a symmetrical structure of communication is positively related to infant mental and psychomotor development and secure attachment status. Asymmetrical and unilateral co-regulation patterns and unengaged behaviours correlated negatively with infant development; a unilateral pattern was also related to an insecure style of attachment.

### Findings: Developmental outcome

This section is an attempt to categorize infant/child developmental outcomes related to early mother-infant interaction quality according to the explored developmental domains. We allocated significant results from selected articles into three main categories of infant functioning and developmental outcomes: (a) physiological, (b) socio-emotional, and (c) cognitive.

#### Physiological development

In this section, we summarize the evidence for the relationship between early interactional quality and biological functions/markers of development on the basis of neuroimaging research, studies on hormonal fluctuations, autonomic nervous system activity changes, or eating problems.

#### Neurodevelopment

Some authors tried to find a way to measure neurological outcomes relevant to the quality of early mother-infant interactions. Bernier and colleagues [22] measured infants 1-minute resting frontal EEG power, which, according to the literature, may be a strong indicator of brain development during infancy. Researchers explored the associations between quality of maternal behaviour during early interactions, infant’s resting EEG power, and increase of resting EEG power at three points: at the infant’s age of 5, 10, and 24 months. Apparently, higher quality maternal care predicted higher frontal resting EEG power at 10 and 24 months, as well as increases in power between 5 and 10 months, and between 10 and 24 months.

Sethna [25] examined infants’ dimensions and brain volumes at the age of 4 months using MRI neuroimaging and correlated it with several maternal and infant behaviours during interaction. Their results showed that infants’ total grey and white matter volume was positively correlated with maternal affect in early interactions (r = 0.33, *p* = 0.042), and cerebral-spinal fluid (CSF) volume correlated negatively with it (r =−0.33, *p* = 0.039), whereas in the case of maternal negative affect (i.e., depressive-like expressions) opposite correlations were observed. However, these associations did not survive correction for multiple testing in further analyses. In addition, positive correlations were found between subcortical infants’ grey volumes and maternal sensitivity in early interactions (r = 0.54, *p* < 0.001), which this time survived correction for multiple comparisons.

#### Basal cortisol level

In the articles we collected, there were also examples of exploring correlation between quality of parent-infant early interactions and physiological outcomes. Tu and colleagues [26] in their research examined infant levels of basal cortisol. According to current knowledge, high levels of this hormone can contribute to the risk of impaired neurodevelopment. The authors succeeded in proving that in the high-stressed group of mothers, higher quality of interactional behaviour correlates with lower infant basal cortisol level, both measured at 8 months CA. The same association was not found in the group of low-stress mothers.

#### Heart rate variability

Zeegers and colleagues [33] registered infants’ autonomic, physiological regulation capabilities controlled by parasympathetic nervous system (PNS) activity by measuring high-frequency (HF) heart rate variability (HRV). The HRV baseline index (mean value over time), assessed in a patient’s resting state, provides information about an individual’s general capacity for emotion regulation. A decrease of the HRV level during and after situational external stimulation can be regarded as an index of the affective arousal regulation. In their research, the stranger approach protocol was used in order to explore HRV decrease after stressful situations. In this task, a male stranger interrupted parental interaction by coming in, talking, and picking up the infant for 30 seconds. Authors correlated both variables (baseline HRV and HRV decline) with maternal and paternal MM and caregiving quality. They found strong evidence that a higher baseline HRV at 12 months of an infant’s age was associated with higher levels of mother’s and father’s MM, and a lower baseline HRV with parents’ poorer MM, independently. Moreover, infants’ larger HRV decline after distress could be predicted by mothers’ appropriate mind-related comments at 4 months and fathers’ non-attuned mind-related comments at 12 months.

#### Feeding/eating difficulties

Different infants’ physiological functioning aspects were observed in a study by Silberstein and colleagues [18]. They explored children’s feeding difficulties in the late infancy, as a physiological outcome related to the early interactional qualities measured in the postnatal period, prior to hospital discharge. At 12 months of age, researchers performed direct observations of feeding interactions, coupled with interviews with participating mothers. Feeding difficulties were associated with maternal interactional features in the neonatal period: higher intrusiveness, less affectionate touch and gaze, more gaze aversion, lower adaptability, and with the infant’s early interactional behaviours, i.e., lower involvement and greater withdrawal.

### Socio-emotional development

#### Temperamental profile and self-regulation

Temperament, defined as “individual differences in emotional, motor, and attentional reactivity (...) and self-regulation processes (…)” [72,73], is often considered an important infant contribution to the quality of their interactions with an adult. At the same time, temperament is, to a certain degree and via complex mechanisms, shaped by an infant’s interactions with the environment, and as such, can also be considered in terms of potential outcomes.

Across studies collected in this review, several well-established tools were used to measure infants’ and children’s temperament as a developmental outcome, starting with a gold standard, the Infant Behavior Questionnaire-Revised (IBQ-R) [74]. Van de Weijer-Bergsma and colleagues [32] used this questionnaire in order to explore the correlation between infant self-regulation and maternal parenting behaviours in early interactions. They did not find significant interdependence; however, other researchers were more successful.

In Costa’s and Figueiredo findings [44], IBQ-R scores representing mothers’ perception of infant high pleasure (IC95% [1.54, 2.05]; *p* = .042) and infants’ smile (IC95% [−2.04, −.56]; *p* = .038) were lower when the quality of early mother-infant interaction was categorized as a poor one. Again, when mother-infant interaction was rated as poor, mothers’ perceptions of infant activity level was higher (IC95% [−1.56, .−54]; *p* = .043).

Davis and colleagues (2018) [15] showed that scoring on the orienting/regulation dimension of IBQ-R can be related to the maternal behavioural pattern, i.e., a degree of predictability. Infants at the end of their first year of life showed poorer regulation capacities if their mothers were more unpredictable in early interactions (there were two cohorts in the study: the Turku Cohort: r = −.28, *p* < .01; the Irvine Cohort: r = −.16, *p* < .05). In this study, the unpredictable maternal behaviours presaged a poorer value of self-control over time in the follow-up covering three time points (5, 6.5, and 9 years of age). To reduce the possibility of maternal bias in reporting infants’ self-regulation abilities, Davis and colleagues also implemented the Children’s Behavior Questionnaire (CBQ) [75] and the Temperament in Early Childhood Questionnaire (TMCQ) [15] and performed direct self-control evaluation via Ericksen’s Flanker task [76], which measures the child’s response inhibition. Those diverse child self-control measures correlated with each other over time.

Another researcher who applied IBQ-R in order to assess infant temperament and correlate it with the quality of maternal interaction was Crawford [28], who explored infants’ attentional capabilities. She found that perceptual sensitivity, which relates to the infant’s ability to detect slight, low intensity stimuli in the environment, was positively related to the maternal sensitivity/responsivity (original terminology of the author) in early interactions.

Milgrom [31] used other parent-completed questionnaires on infant temperament: the Short Temperament Scale for Infants (STSI) and the Short Temperament Scale for Toddlers (STST) [77], trying to explore interdependence between temperament and maternal responsiveness in early interactions, but this relationship was not significant.

Kivijärvi and colleagues [24] discovered an interrelation between maternal sensitivity behaviour and temperamental profile, asking mothers to complete the Revised Infant Temperament Questionnaire (R-ITQ); [78], at 6 months of age and the Toddler Temperament Questionnaire (TTQ) [79] at 12 months of age. Lower levels of maternal sensitivity were found more frequently in the intermediate temperamental clusters of infants, characterized by moderate levels of temperamental features, between the high or low ends of specific temperament dimensions.

Nevertheless, parent-completed questionnaires were not the only method used to assess self-regulation capacities of infants. Some researchers [35] applied a microanalytic approach that included recording infant motor movement, posture, respiration responsiveness, and cry behaviour during and after a painful procedure in NICU and using the Neonatal Distress Pain Related Behavioral Coding Schema (ND-BSC) [80] to code infant reactions. They correlated infants’ pain behaviour self-regulation level with caregivers’ behaviour, clustered in two domains–typical and atypical. Presented findings prove that more atypical infants’ reactions to pain relate to atypical ways of caregiving.

Kivijärvi [24] measured infant temperament, i.e., mood, sociability, and activity, by implementing infant items of the PCERA scales [50]. Mood scores reached concern levels more frequently in the case of less sensitive mothers.

Feldman [37] explored emotion regulation (ER) in children between 3 months and 5 years of age by using several age-specific ER interactional paradigms based on presenting intrusive stimuli, delayed gratification, separation-reunion with caregiver episodes, and other interactional situations that revealed child’s regulatory behaviours and compliance. Infants and children’s ER at five different time points was next correlated with previously measured mother-infant reciprocity in early interactions. Cross-time correlations proved that higher child ER capacities appeared when mother-infant early reciprocity increased.

#### Attachment

The concept of attachment refers to the individual’s internal organization regarding the tie from an infant to a caregiver [81], which can be established as a secure or insecure pattern, depending on early dyadic relationship with the principal attachment figure, i.e., a parent. If the mutual interaction is satisfactory and provides a secure base for exploring the environment, it is likely that a child will develop a secure attachment [82]. Disorganized attachment is characterized by the lack of consistent strategy to organize emotions and behaviours toward the caregiver.

In an experimental setup, infant attachment pattern can be revealed by implementing the protocol of the Strange Situation Procedure (SSP) [83], in which a baby is exposed to a series of separations and reunions with the caregiver and the infant’s reactions can be observed when being left alone, left in the presence of a stranger, or being reunited with a parent. O’Donnell and colleagues [17] implemented this paradigm to assess the security of attachment in preschool-age children and tried to find the interrelation with early maternal behaviour and maternal sensitivity; however, they did not succeed.

Wolke and others [27] applied Main and Solomon’s continuous scale [84] to assess attachment disorganization during the SSP and related it to the maternal sensitivity scores; they found an interrelation between lower maternal sensitivity and infant attachment disorganization (B = −0.57, SE = 0.25, β = −0.21, *p* = 0.019). Evans and Porter [43] administered SSP and explored the interrelation of attachment patterns with early mother-infant co-regulation patterns. Specifically, they found associations between infants’ secure attachment status and symmetrical structure of co-regulation and also between insecure attachment style and higher levels of unilateral mother-infant co-regulation structure. Also, Britton’s findings [38] show that the quality of early dyadic interactions can be predictive of further infant attachment security. The scores on interaction quality scales were higher among dyads with securely attached infants assessed by the Ainsworth Strange Situation [83] (OR = 1.05, 95% CI: 1.01–1.10; *p* < .05).

Ainsworth’s approach to attachment classification was also applied to the observation of preschool age children during a separation-reunion procedure with their mothers [85]. Wazana and colleagues [19] explored the interrelation between early maternal care and the type of attachment at 3 years of the children’s age.

Researchers used Moss’s coding criteria [53] and found that children of mothers who presented less attentive and sensitive early maternal care were those who revealed a disorganized pattern of attachment at the time of mother-child reunion.

#### Affect

Positive and negative affect can be measured either at a baseline or after an exposure to some external stressors. The example of the experimental procedure that can probe the infant affect in response to a standardized interpersonal stressor is the Still Face (SF) procedure [86], which reveals emotional reactions of a child before, during, and after mothers express indifference to their child’s attention-seeking by intentionally maintaining a neutral facial expression, avoiding eye contact, touch, and any interaction with their infant, remaining still, and looking slightly above the infants’ head. Erickson and Lowe [60] coded changes of affect when applying the SF paradigm and related it to the maternal responsiveness level measured before and after the SF procedure. It appeared that higher maternal responsiveness was highly correlated with infant positive affect (r = .70, *p* < 0.001 before SF; r =.64, *p* < .001 after SF). However, no significant correlation with infant negative affect could be found.

#### Social development and adaptation

Parent-child relationships may have a crucial role in building social competences and prosocial behaviours, or may contribute to the early onset of conduct problems (CPs), such as aggression or oppositionality [86]. Feldman [41] found a positive interrelation between a child’s behavioural adaptation measured with the Child Behavior Checklist (CBCL) [88] and mother-infant affective states’ synchrony during early interactions. Feldman also revealed that this kind of synchrony contributes to the development of dialogical empathy in adolescents, assessed during laboratory-driven observation of conflict discussions with their mothers. Dialogical empathy reflects the child’s ability to see other people’s perspective and adapt their view accordingly, discuss their issues in a dialogical manner, and raise adaptive solutions and concern about others’ feelings. The effect of early parent-child reciprocity on empathy was also confirmed in another Feldman study [37] in a group of 10-year-olds In 2010, Feldman [23] proved that a lower level of adolescents’ adaptation measured with the CBCL and the Child Depression Inventory (CDI) [89] was correlated with poorer dyadic reciprocity in early mother-infant interactions. Moreover, Feldman and colleagues [16] assessed a child’s social behaviour at 3 and 13 years of age during interactive events with different partners: mother, father, and a best friend. They correlated it with the indicators of social reciprocity measured at 5 months of age during early caregiver-infant interactions, using the Social Reciprocity composite of CIB manual [65]. The CIB scores represented types of behaviours when partners were engaged in give-and-receive interactions, shared activity, communication, requests, when they were sensitive to each other’s verbal and non-verbal cues, adapted to each other’s needs, or just reflected moments when interaction was fluent and rhythmic. In the preschool period, children’s social competences, aggression, and prosocial behaviour were assessed via the protocol of structured play. In adolescence, dialogical skills were rated during conflict discussion with a partner. Statistical analysis revealed that social reciprocity in early interactions was related to child’s social competences, aggression, and prosocial behaviour at preschool age, as well as to childrens’ dialogical skills in adolescence. It also showed that social competence and lower aggression in preschool age, which shaped dialogical skills in adolescence, were also predicted independently by early maternal and paternal reciprocity.

Other research groups point out that negative parenting in infancy [36], which involves parental expression of anger, frustration, and disgust, can predict conduct problems measured in 5–6-year-olds, using the CBCL [90] and the Teacher Report Form [91]. Shah and colleagues [21] proved that experiencing more negative parenting during the first two years of life predicts externalizing behaviour problems, measured at the age of 3 years using CBCL 1½–5.

## Cognitive development

Here we present studies that measured diverse developmental outcomes connected to cognitive functions, from the general IQ level of children to their crystalized intelligence, executive functions, and particular cognitive abilities, such as language development, communication skills, attention, etc.

### General measures of cognitive development

#### Mental/psychomotor development

Evans and Porter [43] measured infant mental and psychomotor development using the Bayley Scales of Infant Development (BSID II) [92] and found a positive correlation between MDI (mental developmental index) and PDI (psychomotor developmental index) scores at 9 months of age and symmetrical structure of dyadic interaction at 6 months postpartum (r = .44, *p* < .001, r = .21, *p* < .06, respectively). A lower MDI score was related to the asymmetrical (r = −.28, *p* < .01) and unilateral (r =−.37, *p* < .001) patterns of interaction. Moreover, the lower PDI results were linked to the unengaged mutual behaviours of interactive partners (r = −.27, *p* < .05). Poehlmann and Fiese [39] also used BSID II in order to assess infants’ developmental abilities. Their results show direct interrelation between Bayley’s mental scales and the quality of parent-infant interaction. MDI scores, measured at 12 months of age, were higher when infants’ early interaction with mothers (6 months postpartum) was more reciprocal, affectively positive, and engaging.

#### IQ

Milgrom and colleagues [31] applied the Wechsler Preschool Primary Scale of Intelligence (Revised) (WPPSI-R) [93] in two groups of infants, the clinical one, whose mothers were depressed at 6 months postpartum and the control one, whose mothers were healthy. Children of mothers with postpartum depression had significantly lower full-scale IQ measured in toddlerhood, as well as poorer performance on particular Wechsler’s subscales like geometric design and arithmetic. Statistical modelling revealed a large mediating effect of maternal responsiveness level explaining the above-mentioned correlation, since responsiveness is the interactional quality strongly associated with postpartum depression. Another example of the interrelation between maternal parenting and IQ was presented by Shah with co-workers [21]. Their results show a positive correlation between quality of maternal parenting and general IQ score of a very preterm infant (VPI: < 30 weeks), measured with the Abbreviated Battery IQ Scale (ABIQ) from the Stanford-Binet Intelligence Scales, 5th edition [94]. Feldman [41] used the same tool to measure the verbal IQ of preschool and school-aged children and found its interrelation with mother-infant synchrony of affective states across the first year postpartum.

#### Executive functioning

Van de Weijer-Bergsma and colleagues [32] correlated early interactional experiences of infants with the development of executive functions (EFs). EFs are differently defined in the literature, but in general this term covers a number of higher-order cognitive processes that allow one to perform goal-oriented behaviour [95]. In the above-mentioned study, authors measured working memory, inhibitory control, and attention control via performance on so-called reversal tasks, namely, the A-not-B task [96]. The speed of developmental progress in EF measured at three time points via the A-not-B task was positively correlated with maternal level of directiveness assessed in an interactional context (r = 0.54, one-sided *p* < 0.05). An attempt to correlate developmental change in EF with maternal sensitive responsiveness did not result in statistically significant findings.

#### Focused attention

Tu and colleagues [26] concentrated on a different infants’ cognitive competence, namely, focused attention. Tu applied Lawson and Ruff’s [97] scoring method to mark out the attentional processes during videotaped interactional mother-infant toy exploration and related it to the interactivity level in maternal behaviour. The results differed according to the index of parenting stress. In the high-stress group of parents, the higher interactive behaviour was associated with lower quality of focused attention, and, in contrast, in the low-stress group of parents, the higher interactive behaviour had a positive correlation with focused attention.

#### Language and communication

Stolt and colleagues[40 explored the association between early maternal interactions and child early-language development. With children at the age of 6 months, they used the Checklist for the Development of Early Vocalizations (CDEV), and at 12 and 24 months, they tested lexical development using the Finnish version of the MacArthur Communicative Development Inventory (CDI; FinCDI). The results showed a positive correlation between linguistic competences and several determinants of interactional quality at 6 months CA, i.e., dyadic mutuality, flatness, disorganization, and tension. Milgrom and colleagues [29] explored the interrelation between interactional quality in the mother-newborn relationship and the development of infant communication. She measured child early communication abilities at the age of 6 months by asking mothers to fill out the Infant–Toddler Checklist of the Communication and the Symbolic Behavior Scales Developmental Profile (CSBS DP Infant–Toddler Checklist) [98]. Milgrom proved that higher scores in symbolic composite of CSBS were found in infants whose mothers were assigned to the experimental group, which received the MITP parent training program, and in consequence, presented higher maternal sensitivity, more positive affect toward infants, and more synchronic dyadic interactions. Furthermore, Newnham and colleagues [30] found significant differences in communication competences, measured by the Ages and Stages Questionnaire (ASQ) [99], between analogical groups of two-year-old children with diverse levels of maternal responsiveness in early interactions. The correlation was positive. Symbolic behaviour was also measured in free play at 12 and 18 months by Giovanelli [34] using the Coding System of Symbolic Play [100]. The infants’ maturity level of symbolic play at 18 months of age was positively correlated with higher scores of mothers’ MM regarding appropriate comments in the subcategory of emotions. The average level and length of symbolic play at 12 months of age was also positively related to the appropriate mind-related comments in subcategories of desires and cognitions.

## Discussion

The quality of early parent-infant interactions plays a central role in shaping infant developmental outcomes, encompassing socio-emotional, cognitive, behavioural, and neurobiological domains [3,4,13]. The research in this area primarily focused on caregiver-related factors, i.e., maternal sensitivity, responsiveness, or affect [3,6,8,9], while more current perspectives increasingly highlight the dynamic, reciprocal nature of social exchanges, highlighting how an infant’s own behavioural signals, affective states, and regulatory capacities actively shape the interactional flow [10,12,13] These models point to interactional contingency [5] and co-regulation [11] as core mechanisms through which early relational experiences relate to developmental outcome.

In this systematic review, we point to different aspects of the quality of early interactions between mother and infant related to the particular developmental outcomes of an infant. In most of the studies the unidirectional perspective on building an early interaction was applied, so just the maternal caregiving quality or her behavioural features were measured and related to the children’s developmental outcomes. There are studies that connect early maternal behaviour with socio-emotional and cognitive development, as well as physiological and affective regulation capacities of a child. A generalized score of early maternal caregiving quality was related with children’s basal cortisol level, resting frontal EEG power, self-regulation behaviour, focused attention, social reciprocity in relationships, behaviour problems, and IQ. Maternal level of early sensitivity was significantly related to the child’s temperament, baseline positive affect, pattern of attachment, cognitive competences, communication abilities, and even a volume of subcortical grey matter. Maternal directiveness was correlated with a faster acquisition of executive functions. Mothers’ early intrusiveness was important in developing feeding difficulties in older infants. Maternal affect toward infants was related with children’s brain development, namely, their total grey and white matter volume, CSF volume, as well as feeding difficulties and conduct problems. Parental MM was related to child’s symbolic play development and physiological regulation, namely, baseline HRV and HRV decline after distress. Furthermore, the estimated probability of maternal behaviours was connected to the level of infants’ self-regulation abilities.

The unilateral approach to measuring interaction quality has many advantages. The research design is rather simple; many methodological tools based on videorecorded interactions are well established and standardized. However, using just a unilateral perspective narrows our view on the real nature of parent-infant interaction, since it is known that many aspects of interactional quality are driven bidirectionally, by both maternal and infant behavioural clues.

The approach that captures mutual mother-infant interactive behaviours, measuring dyadic interactional features or communication structure, gives a more exact and naturalistic view of the quality of early relationships. Unfortunately, it was applied in only 11 studies out of the 30 we selected. Some of the researchers who decided to measure bidirectional processes in interactions used overall scales reflecting dyadic factors of interaction. They correlated them with children’s temperamental profiles, patterns of attachment, and linguistic competences. Dyadic reciprocity, as a particular interactional feature, was found to be correlated also with mental development, emotion regulation, aggression, social skills, and empathy. Some other methods captured and evaluated the symmetrical co-regulation sequences during early mother-infant interaction, which appeared to be positively correlated with the infant’s secure attachment and better mental and psychomotor development. Furthermore, the synchrony of reactions between mother and child was related to the children’s empathy, behaviour adaptation, and IQ.

**Figure 2.**
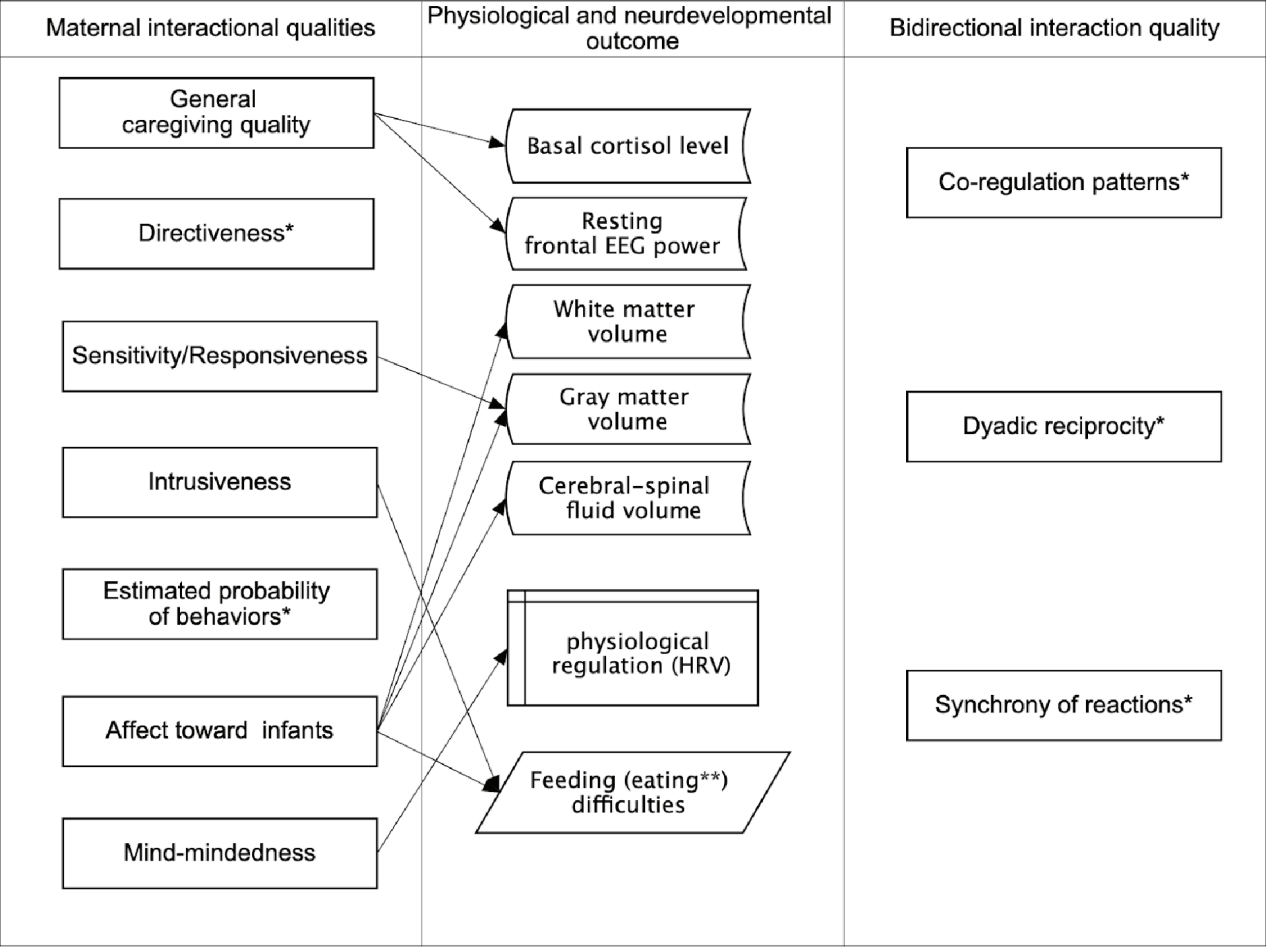
Significant direct interrelations between the level of early mother-infant interaction quality and physiology-related developmental outcomes found within the scope of articles included in this review. *no studies which include correlation with physiological or neurodevelopmental outcome. **eating difficulties according to the current terminology.

**Figure 3.**
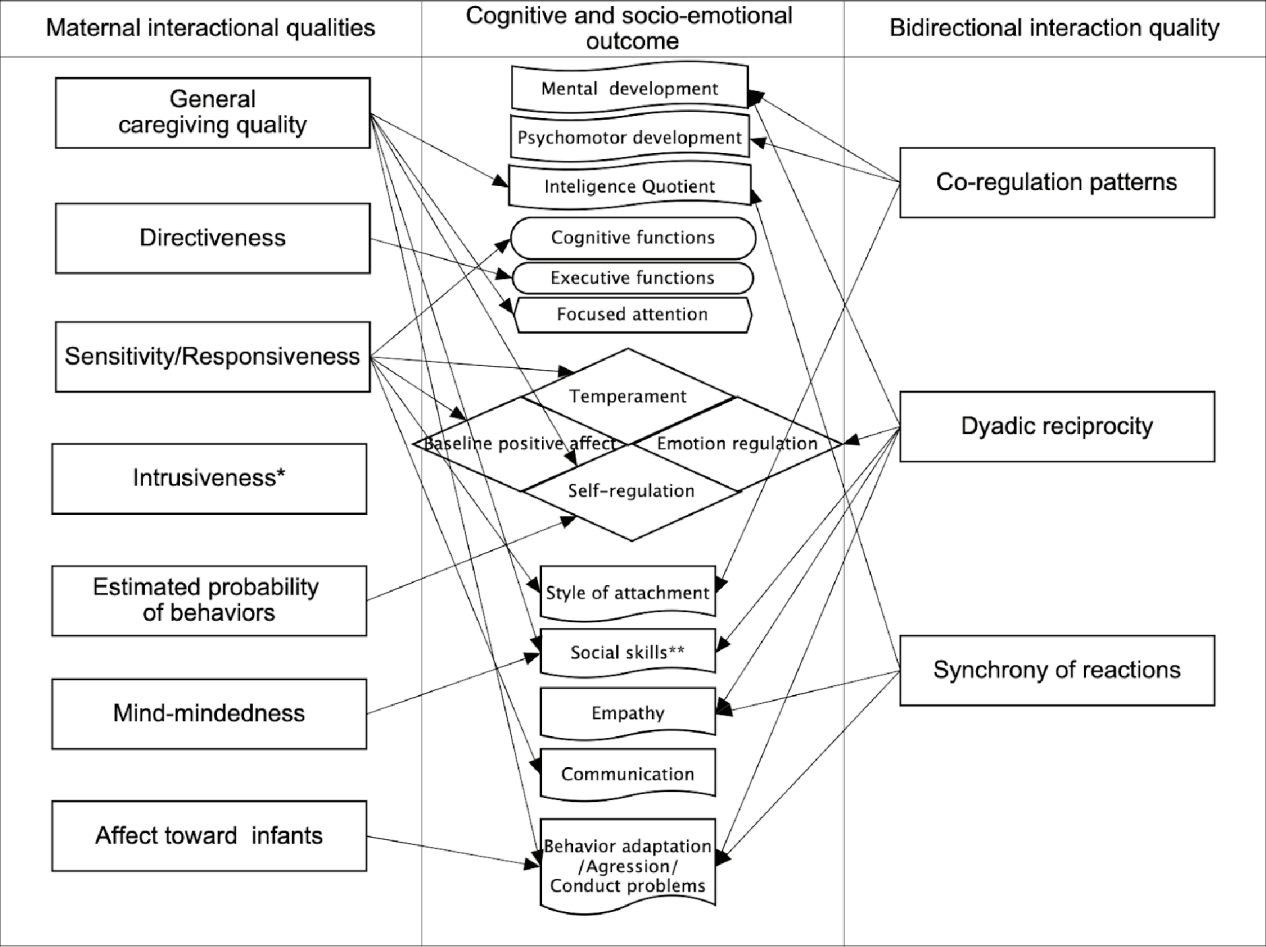
Significant direct interrelations between the level of early mother-infant interaction quality and cognitive-related developmental outcomes found within the scope of articles included in this review. *no studies which include correlation with cognitive or socio-emotional outcome. **Social skills include three different variables found in articles: social skills, social reciprocity and symbolic play development.

The above-mentioned correlations are visualized in Fig. 2, which represents the interrelation between early interactional factors and children’s physiological and neurodevelopmental outcomes, and in Fig. 3, in which interactional variables are related to children’s cognitive and socio-emotional developmental outcomes.

Among the collected papers, we can see the prevalence of research that measures unidirectional (maternal) qualities in interactions over evaluating bidirectional/dyadic characteristics of communication. Moreover, the review reveals a dominance of studies that assess cognitive and socio-emotional developmental achievements of children over those that evaluate biological development-related indices or physiological functioning of a child. As we can see in Fig. 3, in the scope of our review results, there is a complete lack of the research that integrates a bidirectional approach to measuring interaction quality and children’s physiological or neurodevelopmental outcome.

## Limitations

This review points to a number of potential effects that the quality of early parent-infant interaction can have on the behavioural and biological outcomes of a child. The inclusion criteria for articles were restrictive in order to be in line with our research questions. As our aim was to focus on measuring early interactional quality, we limited our analyses to the studies that assessed mother-infant interactions up to 8 months of infants’ age. This could be view-limiting on other methods of interaction quality evaluation. The developmental outcomes in the collected papers, on the other hand, were not restricted with exclusion criteria, but usually referred to some particular domains or competences and did not reflect the holistic developmental assessment of a child.

Longitudinal exploration of the effects of early interactions makes the experimental setting difficult. Early videorecording set up at NICUs and follow-up home-based meetings in such kinds of studies usually make the attrition rate very high. This fact significantly affects the studies’ final sample size, and it is the reason why many performed data analyses were just correlation-based and could not present the direction of associations.

It should be also noted that many of the studies included in the review were conducted in NICU settings. This clinical context creates certain constraints on the generalizability of the findings into different populations, since mother-infant interactions observed in NICUs may be significantly biased by elevated stress levels, specific medical protocols, and interruptions in the continuity of caregiving. Such environmental factors alter the natural course of early interactions and influence infant developmental trajectories.

What is more, we noticed inconsistency in measuring co-factors across studies. The omitted correlates that can play a role in modulating interdependencies between interaction quality and developmental outcomes bias the comprehensive understanding of the processes under study. The important maternal factor that was not incorporated in the majority of studies, but can influence early mother-infant interactions, is socio-economic status (mothers’ education level, social support, and financial resources). Studies indicate that mothers who face low income or lower social support or education levels are less likely to present sensitive and responsive behaviours toward their infants [101,102,103,104]. Conversely, higher maternal education, community support, and greater economic stability are associated with a higher quality of interactions and were identified as protective factors for child development.

Another limitation could be the lack of integration of sociocultural aspects in most of the research included in our systematic review. The mother-infant interaction processes are influenced by social structures and are connected to population differences and culturally defined norms of motherhood. What constitutes a “good mother” is socially related and can differ between cultures and social classes. Gillies (2006) [103] highlights how marginalized working-class mothers have often been stigmatized as “bad mothers,” and middle-class mothers’ behaviour often sets the standards by which parenting practices are judged. In some research, there were categories used such as “typical” and “atypical” maternal behaviours for measurement purposes, but we acknowledge that such classifications carry normative assumptions. What is considered “typical” in one cultural context may be viewed differently in another. For example, some non-Western cultures prioritize close physical connectedness between mother and infant (through co-sleeping, prolonged breastfeeding, baby-wearing), whereas Western norms tend to encourage earlier infant autonomy [105]. This means that our view on certain behaviours as standard or deviant can be limited, as they are more accurate in Western cultural contexts, rather than being some universally applicable norms. Additionally, mothers often perform significant emotional labour in order to be congruent with social expectations [106]. Mothers can intentionally show “good” mother behaviour that is culturally “appropriate”, while suppressing any “inappropriate” emotions. This process can be invisible and omitted in research; however, it can imply that maternal responsiveness, warmth, patience, or synchrony is not purely instinctual, but may be molded by societal norms and expectations.

To sum up, the above-mentioned factors omitted in the reviewed studies could bias the findings that this systematic review collected. Thus, future research should integrate those important lacunae to provide a more nuanced, comprehensive insight into the interplay between early interaction quality and infant developmental trajectories.

## Future directions

First of all, an attempt should be made to clarify and unify wide and inconsistent terminology within the discussed scientific domain; there is often no unique term that encompasses overlapping meanings, which clearly is a result of approaching the subject from diverse conceptual and theoretical frameworks and academic disciplines.

The main findings of this review proved that both unidirectional and bidirectional composites of early parent-infant interactions can play a role in building children’s strong cognitive and social competences and thereby implicitly predict their well-being and further adjustment in adult life. An attempt should be made to identify, as soon as possible, mother-infant dyads at risk of less supportive interactive patterns in order to provide them with all necessary support. Some NICUs have already designed and implemented interventions in order to improve parental interactional quality by enhancing their sensitivity level and reducing their stress [107]. Among collected papers in this review, there are two examples of NICU-based therapeutic programs for parents [29,31], both constructed on the basis of the Mother–Infant Transaction Program (MITP) [57]. They consist of techniques that focus on the improvement of parenting efficacy and sensitivity in early interactions. The above-mentioned interventions proved to have a beneficial effect on later infant development, namely, communication abilities, cognitive skills, and temperamental profile. Thus, further investigations in the field are needed, since detecting early interactional factors that may contribute to children’s developmental outcomes has many implications for clinical practice and therapy. This way, diverse programs can be designed and applied in NICUs, in order to support positive mother-infant early interaction and enhance the youngest individuals’ development from the very first moments of their life. Such programs and recommendations should be carefully designed to support parents and infants in their early interactions, especially in contexts and situations when the risk of interactive difficulties may be higher (NICU parents, parents with symptoms of depression, etc.). At the same time, it should be emphasized that there exists a wide diversity of parent and infant behavioural repertoire in interactions that are mutually dependent and can be elaborated in time, resulting in more reciprocal patterns. Every attempt should be made to avoid stigmatization and pathologizing less frequent or less typical, but still supportive, interactional patterns. Furthermore, little can be said about the timing and possible windows of opportunity for interventions that could promote infant development by increasing the quality of parent-infant early interactions. Only a few studies in this review were randomly controlled trials or represented an experimental (or at least quasi-experimental) design. When would be the best time to intervene, for how long, and at what level? There exists some evidence that, at least in the case of mothers and preterm infants, initial difficulties in interactions may fade away with time [108]. Can intervention enhance or rather, does it interfere with this process of “self-correcting tendency”? All these questions are urgent and require further studies.

The last two decades of research, in line with the papers included in the review, show an emerging trend for searching new analytical methods of measuring dyadic qualities in early interactions that can be related to future children’s development. Many of these approaches became available thanks to advances in data computing techniques. Exploring co-regulation patterns between mother and child or measuring a synchrony of their mutual reactions during interaction is a promising way to acquire a more comprehensive understanding of early interactional factors that matter for child development. However, across the selected literature, we found a complete lack of studies exploring the interrelation between bidirectional qualities of early interactions and physiological and brain-related children’s outcomes (see Fig 2). There is definitely a crucial need for more research on the above-mentioned matter. The aim should be to capture multiple trajectories of how early interactional qualities coupled with children’s individual biological and temperamental features, under the influence of particular environmental contexts, can lead to better developmental outcomes. The goal should be to create the closest possible model of early experience, seen as a complex system of interrelations between the culturally and socially situated interactional input and infant’s characteristics, in order to identify the changeable factors that can either harm or promote optimal development throughout childhood.

## Conclusions

Interactional quality in early relationships with parents is a construct defined diversely in the literature and can be evaluated on different dimensions. In this review, we present research methodologies on interactional quality deriving from several available theoretical frameworks. We also analysed related developmental outcomes from numerous developmental domains that represent children’s particular set of competences. With evidence from the review, we can draw a clear conclusion that the quality of early mother-infant interaction is a very important factor promoting the development and future life of a child. However, the evidence is rather patchy and does not provide a comprehensive view on the complexity of the matter, considering cascadic, multidirectional interdependencies between the mother-infant dyad and the environment. The goal for future studies should be to find possible ways of understanding the mentioned processes, as a complex system of probable interrelations, and look for both blockades and opportunities in the process of natural mother-infant attunement as potential targets for developmentally supportive, time- and context-sensitive interventions.

### Key points

1. Unilateral interactive maternal characteristics, including maternal sensitivity, directiveness, intrusiveness, affect, and MM correlate with children’s cognitive, socio-emotional, and neurophysiological outcomes.
2. Bidirectional dyadic interaction quality measures, including synchrony, reciprocity, and co-regulation, correlate with children’s social competencies, emotional regulation abilities, and psychomotor development.
3. There is a notable research gap regarding studies that integrate bidirectional measures of mother-infant interaction quality with the physiological or neurodevelopmental of the child.

## Appendix 1 Results of searching in databases

***SCOPUS***

Trial 1: (TITLE-ABS-KEY (“„preterm infant*” OR neonate* OR “preterm child”) AND TITLE-ABS-KEY (“parent-child interaction*” OR “mother-child intervention*” OR “autonomic synchrony” OR “maternal early intervention*” OR “parental early intervention*” OR “parent-infant synchrony” OR “early intervention* with parents” OR “mother-child attunement” OR “parental responsiveness” OR “biobehavioral synchrony” OR “concordance of biological rhythms” OR “parent reciprocity”) AND TITLE-ABS-KEY (“neurodevelopmental outcome*” OR “socio-emotional outcome*” OR “cognitive functioning” OR “cognitive function*” OR “brain development” OR “neurobehavioral outcome*” OR “pain reactivity” OR “autonomic nervous system stability” OR “cognitive outcome*” OR “autonomic stability”))

***Results: 7***

Trial 2: (TITLE-ABS-KEY (preterm OR infant* OR neonate*) AND TITLE-ABS-KEY (“parent-child interaction*” OR “interaction* quality” OR “relation* quality” OR “autonomic synchrony” OR “maternal intervention*” OR “early intervention*” OR “parent-infant synchrony” OR “attunement” OR “parental responsiveness” OR “early relation*” OR “maternal relation*”) AND TITLE-ABS-KEY (“early outcome*” OR “neurodevelopmental outcome” OR “neurobehavioral outcomes” OR “stressful exposure” OR “pain reactivity” OR “autonomic nervous system” OR stability OR “early temperament*” OR “early social”))

***Results: 387***

Trial 3 (TITLE-ABS-KEY (newborn OR preterm OR premature OR neonate*) AND TITLE-ABS-KEY (“maternal care” OR “parent-child interaction*” OR “parent-infant interaction*” OR “mother-child interaction*” OR “mother-infant interaction*” OR “interaction* with parent*” OR “interaction* with mother” OR “interaction* quality” OR “relation* quality” OR “autonomic synchrony” OR attunement OR “parent-infant synchrony” OR “maternal intervention*” OR “parental responsiveness” OR “maternal responsiveness” OR “reciprocity” OR “early relation*” OR “maternal relation*”) AND TITLE-ABS-KEY (neurodevelopment OR “neurodevelopmental outcome*” OR “neurobehavioral outcome*” OR “early outcome*” OR “early infancy” OR “stress reactivity” OR “pain reactivity” OR “autonomic nervous system” OR “parasympathetic nervous system” OR stability OR “early temperament*” OR “physiological response” OR “physiological activity” OR nipe))

***Results: 239***

**Web of Science**

Repeated Trial 3 from above

***Results: 116***

**PubMed**

Trial 4: (newborn[Title] OR preterm[Title] OR premature[Title] OR neonate)[Title] AND (“maternal care”[Title] OR “parent-child interaction”[Title] OR “parent-infant interaction”[Title] OR “mother-child interaction”[Title] OR “mother-infant interaction”[Title] OR “autonomic synchrony”[Title] OR attunement[Title] OR “parent-infant synchrony”[Title] OR “maternal intervention”[Title] OR “parental responsiveness”[Title] OR “maternal responsiveness”[Title] OR reciprocity[Title] OR “early relation”[Title] OR “maternal relation”)[Title] AND (neurodevelopment[Title] OR “neurodevelopmental outcomes”[Title] OR “neurobehavioral outcomes”[Title] OR “early outcomes”[Title] OR “early infancy”[Title] OR “stress reactivity”[Title] OR “pain reactivity”[Title] OR “autonomic nervous system”[Title] OR “parasympathetic nervous system”[Title] OR stability[Title] OR “early temperament”[Title] OR “physiological response”[Title] OR “physiological activity”[Title] OR nipe)[Title])) OR ((newborn[Abstract] OR preterm[Abstract] OR premature[Abstract] OR neonate) [Abstract] AND (“maternal care”[Abstract] OR “parent-child interaction”[Abstract] OR “parent-infant interaction”[Abstract] OR “mother-child interaction”[Abstract] OR “mother-infant interaction”[Abstract] OR “autonomic synchrony”[Abstract] OR attunement[Abstract] OR “parent-infant synchrony”[Abstract] OR “maternal intervention”[Abstract] OR “parental responsiveness”[Abstract] OR “maternal responsiveness”[Abstract] OR reciprocity[Abstract] OR “early relation”[Abstract] OR “maternal relation”)[Abstract] AND (neurodevelopment[Abstract] OR “neurodevelopmental outcomes”[Abstract] OR “neurobehavioral outcomes”[Abstract] OR “early outcomes”[Abstract] OR “early infancy”[Abstract] OR “stress reactivity”[Abstract] OR “pain reactivity”[Abstract] OR “autonomic nervous system”[Abstract] OR “parasympathetic nervous system”[Abstract] OR stability[Abstract] OR “early temperament”[Abstract] OR “physiological response”[Abstract] OR “physiological activity”[Abstract] OR nipe)[Abstract])) OR ((newborn[Body - Key Terms] OR preterm[Body - Key Terms] OR premature[Body - Key Terms] OR neonate)[Body - Key Terms] AND (“maternal care”[Body - Key Terms] OR “parent-child interaction”[Body - Key Terms] OR “parent-infant interaction”[Body - Key Terms] OR “mother-child interaction”[Body - Key Terms] OR “mother-infant interaction”[Body - Key Terms] OR “autonomic synchrony”[Body - Key Terms] OR attunement[Body - Key Terms] OR “parent-infant synchrony”[Body - Key Terms] OR “maternal intervention”[Body - Key Terms] OR “parental responsiveness”[Body - Key Terms] OR “maternal responsiveness”[Body - Key Terms] OR reciprocity[Body - Key Terms] OR “early relation”[Body - Key Terms] OR “maternal relation”)[Body - Key Terms] AND (neurodevelopment[Body - Key Terms] OR “neurodevelopmental outcomes”[Body - Key Terms] OR “neurobehavioral outcomes”[Body - Key Terms] OR “early outcomes”[Body - Key Terms] OR “early infancy”[Body - Key Terms] OR “stress reactivity”[Body - Key Terms] OR “pain reactivity”[Body - Key Terms] OR “autonomic nervous system”[Body - Key Terms] OR “parasympathetic nervous system”[Body - Key Terms] OR stability[Body - Key Terms] OR “early temperament”[Body - Key Terms] OR “physiological response”[Body - Key Terms] OR “physiological activity”[Body - Key Terms] OR nipe)[Body - Key Terms])

***Results: 103***

**Cochrane Library**

Repeated Trial 3 from above

***Results: 41***

**EBSCO**

Repeated Trial 3 from above

***Results: 191***

**Google Scholar**

Mixed phrases and words from scope: WHO-WHAT-HOW

***Collected results: 671***

***The final number of collected papers: 1755***

## Appendix 2 Quality assessment of studies included in the systematic review.

(Quality Assessment Tool for Quantitative Studies: Armijo-Olivo, Stiles, Hagen, Biondo, & Cummings, 2012; Thomas, Ciliska, Dobbins, & Micucci, 2004)

**Table:**

**No.**	**Author/Year**	**Selection Bias**	**Study Design**	**Confounders**	**Blinding**	**Data Collection Method**	**Withdrawals and Dropouts**	**Global Rating**
1.	A. Bernier, S. D. Calkins, M. A. Bell (2016)	Moderate	Moderate	Strong	Moderate	Strong	Weak	Moderate
2.	J. R. Britton, H. L. Britton, V. Gronwaldt (2006)	Moderate	Moderate	Moderate	Moderate	Strong	Weak	Moderate
3.	R. Costa, B. Figueiredo (2011)	Strong	Moderate	Strong	Moderate	Strong	Weak	Moderate
4.	J. S. Crawford (2004)	Moderate	Moderate	Moderate	Moderate	Strong	Moderate	Strong
5.	E. P. Davis et al. (2018)	Moderate	Moderate	Strong	Moderate	Strong	Moderate	Strong
6.	S. J. Erickson, J. R. Lowe (2008)	Weak	Moderate	Strong	Moderate	Strong	Weak	Weak
7.	C. A. Evans, C. L. Porter (2009)	Weak	Moderate	Strong	Moderate	Strong	Strong	Moderate
8.	R. Feldman (2007)	Moderate	Moderate	Strong	Moderate	Strong	Strong	Strong
9.	R. Feldman (2010)	Moderate	Moderate	Strong	Moderate	Strong	Strong	Strong
10.	R. Feldman et al. (2013)	Weak	Moderate	Strong	Moderate	Strong	Moderate	Moderate
11.	R. Feldman, E. Bamberger, Y. Kanat-Maymon (2013)	Moderate	Moderate	Moderate	Moderate	Strong	Moderate	Strong
12.	R. Feldman (2015)	Moderate	Moderate	Strong	Moderate	Strong	Moderate	Strong
13.	C. Giovanelli et al. (2020)	Weak	Moderate	Strong	Moderate	Strong	Strong	Moderate
14.	M. Kivijärvi et al. (2005)	Moderate	Moderate	Strong	Moderate	Strong	Strong	Strong
15.	M. F. Lorber, B. Egeland (2011)	Weak	Moderate	Strong	Moderate	Strong	Strong	Moderate
16.	J. Milgrom et al. (2013)	Weak	Strong	Strong	Moderate	Strong	Strong	Moderate
17.	J. Milgrom, D. T. Westley, A. W. Gemmill (2004)	Moderate	Strong	Moderate	Moderate	Strong	Moderate	Strong
18.	C. A. Newnham, J. Milgrom, H. Skouteris (2009)	Strong	Strong	Strong	Moderate	Strong	Strong	Strong
19.	K. A. O’Donnell, H. Gaudreau (2014)	Moderate	Moderate	Strong	Moderate	Strong	Weak	Moderate
20.	J. Poehlmann, B. H. Fiese (2001)	Moderate	Moderate	Strong	Moderate	Strong	Moderate	Strong
21.	V. Sethna et al. (2017)	Weak	Moderate	Stong	Moderate	Strong	Strong	Moderate
22.	P. E. Shah, et al. (2013)	Moderate	Moderate	Stong	Moderate	Strong	Strong	Strong
23.	D. Silberstein et al. (2009)	Moderate	Moderate	Strong	Moderate	Strong	Strong	Strong
24.	S. Stolt et al. (2014)	Moderate	Moderate	Weak	Moderate	Strong	Strong	Moderate
25.	M. T. Tu et al. (2007)	Moderate	Moderate	Strong	Moderate	Strong	Weak	Moderate
26.	E. van de Weijer-Bergsma et al. (2016)	Moderate	Moderate	Weak	Moderate	Strong	Strong	Moderate
27.	F. F. Warnock et al. (2016)	Moderate	Moderate	Strong	Moderate	Strong	Moderate	Strong
28.	A. Wazana et al. (2015)	Moderate	Moderate	Strong	Moderate	Strong	Strong	Strong
29.	D. Wolke, S. Eryigit-Madzwamuse, T. Gutbrod (2014)	Strong	Strong	Strong	Moderate	Strong	Strong	Strong
30.	M. A. J. Zeegers et al. (2018)	Weak	Moderate	Strong	Moderate	Strong	Moderate	Moderate
